# Novel Floral Scent Compounds from Night-Blooming Araceae Pollinated by Cyclocephaline Scarabs (Melolonthidae, Cyclocephalini)

**DOI:** 10.1007/s10886-018-1018-1

**Published:** 2018-09-19

**Authors:** Artur Campos D. Maia, Christopher Grimm, Mario Schubert, Florian Etl, Eduardo Gomes Gonçalves, Daniela Maria Do Amaral Ferraz Navarro, Stefan Schulz, Stefan Dötterl

**Affiliations:** 1 Programa de Pós-graduação em Biologia Animal, Universidade Federal de Pernambuco, Recife, 50670-901 Brazil; 2 Institute of Organic Chemistry, Technische Universität Braunschweig, Hagenring 30, 38106 Braunschweig, Germany; 3 Department of Biosciences, University of Salzburg, Billrothstraße 11 and Hellbrunnerstraße 34, 5020 Salzburg, Austria; 4 Department of Botany and Biodiversity Research, University of Vienna, Rennweg 14, 1030 Vienna, Austria; 5 Curso de Ciências Biológicas, Universidade Católica Dom Bosco, Campo Grande, 70790-100 Brazil; 6 Departamento de Química Fundamental, Universidade Federal de Pernambuco, Recife, 50740-560 Brazil

**Keywords:** Preparative gas chromatography, NMR spectroscopy, High resolution mass spectrometry, Floral scents, Volatile organic compounds, Attractants, Beetle pollination

## Abstract

Nocturnal flowering plants often release strong scents to attract their pollinators. Among night active flower visitors are cyclocephaline scarab beetles, which have been demonstrated to respond to uncommon volatile organic compounds released in high amounts by their host plants. In Araceae, the molecular structure of several such compounds is yet to be unveiled. We investigated headspace floral scent samples of *Philodendron squamiferum, Thaumatophyllum mello-baretoanum*, and *Xanthosoma hylaeae* by a variety of approaches, leading to the identification of novel compounds. Dehydrojasmone, (*Z*)-4-methylene-5-(pent-2-en-1-yl)cyclopent-2-en-1-one (**1**), (*Z*)-3-methylene-2-(pent-2-en-1-yl)cyclopentyl acetate (isojasmyl acetate, **3**), and (*E*)-4,8-dimethylnona-1,3,7-trien-5-yl acetate (**4**) had not been previously reported, while full analytical data of the recently described (*Z*)-3-methylene-2-(pent-2-en-1-yl)cyclopentan-1-ol (isojasmol, **2**) are presented here. All these compounds are derived from more common precursors, (*Z*)-jasmone and (*E*)-4,8-dimethyl-1,3,7-nonatriene, likely through biosynthetic “post-processing”.

**Electronic supplementary material:**

The online version of this article (10.1007/s10886-018-1018-1) contains supplementary material, which is available to authorized users.

## Introduction

Angiosperms release a plethora of floral volatile organic compounds (VOCs) (Knudsen et al. 2006), often paramount for effective chemical communication with pollinators (Raguso 2008). Butterflies, moths, bees, bats, and birds, among others, are all well known to respond to several of the components released by flowers and inflorescences of their preferred host plants (Dobson 2006; Kessler and Baldwin 2007). For nocturnal pollinators, the challenging task of locating flowers under dim light conditions often relies on volatile chemical cues (Raguso 2001).

Recent studies about the reproductive ecology of night-blooming Neotropical angiosperms have shown that VOCs derived from a number of different biosynthetic metabolic pathways are implicated with the attraction of cyclocephaline scarabs (Melolonthidae, Cyclocephalini), a diverse group of highly specialized anthophilous insects (Moore et al. 2018). Among such compounds are *cis*-jasmone (**5**), 4-methyl-5-vinylthiazole (Maia et al. 2012), (*S*)-2-hydroxy-5-methylhexan-3-one (Maia et al. 2013), 4-vinylanisole (Dötterl et al. 2012), dihydro-β-ionone (Pereira et al. 2014), methyl 2-methylbutyrate (Gottsberger et al. 2012), and 2-alkyl-3-methoxypyrazines (Maia et al. 2018). Either alone or in simple combinations with other constituents, these VOCs are selectively attractive to species belonging to the genera *Cyclocephala* Dejean, 1821 and *Erioscelis* Burmeister, 1847. It is assumed that their chemical diversity plays a key role in the reproductive isolation and species diversification of several lineages of Neotropical angiosperms, including Annonaceae, Magnoliaceae, and Araceae (Gibernau 2016; Pereira et al. 2014).

A considerable number of quantitatively prominent floral scent constituents that have been trapped in headspace samples of different species of cyclocephaline scarab-pollinated Araceae are yet unidentified. This is for example true for *Taccarum ulei* Engl. & K. Krause (Maia et al. 2013), *Dieffenbachia aurantiaca* Engl. (Etl et al. 2016), and *Xanthosoma* spp. (Milet-Pinheiro et al. 2017), as well as for several species of the diverse *Philodendron s.l.* (over 500 spp. according to Boyce and Croat 2018; Etl and collaborators, unpubl. data). In many cases major constituents in the floral odor of hostplants preferred by cyclocephaline scarabs induce attraction of the beetles. Therefore it seems very likely that these unidentified compounds are involved in selective pollinator attraction as well.

**Fig. 1:**
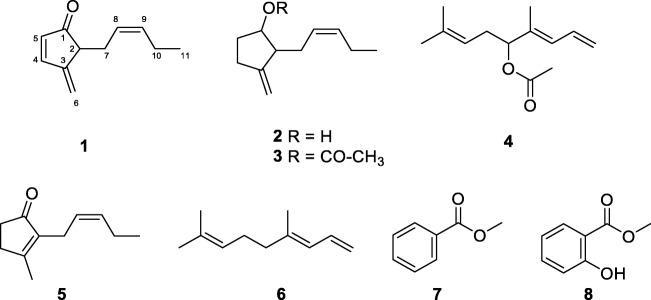
Major floral volatile components of *Philodendron squamiferum*, *Thaumatophyllum mello-barretoanum*, and *Xanthosoma hylaeae* (Araceae). (*Z*)-4-methylene-5-(pent-2-en-1-yl)cyclopent-2-en-1-one (**1**); (*Z*)-3-methylene-2-(pent-2-en-1-yl)cyclopentan-1-ol (**2**); (*Z*)-3-methylene-2-(pent-2-en-1-yl)cyclopentyl acetate (**3**); (*E*)-4,8-dimethylnona-1,3,7-trien-5-yl acetate (**4**); (*Z*)-jasmone (**5**); (*E*)-4,8-dimethylnona-1,3,7-triene (**6**); methyl benzoate (**7**); methyl salicylate (**8**)

In this study, we explored the chemical composition of the floral scent bouquets of two species of *Philodendron s.l., Philodendron squamiferum* and *Thaumatophyllum mello-baretoanum*, and reassessed previously analyzed dynamic headspace samples of *Xanthosoma hylaeae* (Milet-Pinheiro et al. 2017) by GC/MS. Four compounds, **1**–**4** (Fig. 1), present as main constituents in headspace samples of the three studied species, were identified by NMR spectroscopy, either following preparative gas chromatography or by directly subjecting headspace samples to the analysis. Three novel compounds are presented and the identity of another, just recently described without analytical details as a floral scent constituent in Cyclanthaceae (Teichert et al. 2018), is confirmed. All four VOCs are derived from well known semiochemicals (El-Sayed 2018) that are also widespread among cyclocephaline pollinated aroids (Etl et al. 2016; Maia et al. 2012; Milet-Pinheiro et al. 2017), (*Z*)-jasmone (**5**) and (*E*)-4,8-dimethyl-1,3,7-nonatriene (**6**).

## Methods and Materials

### Studied Species

**Fig. 2:**
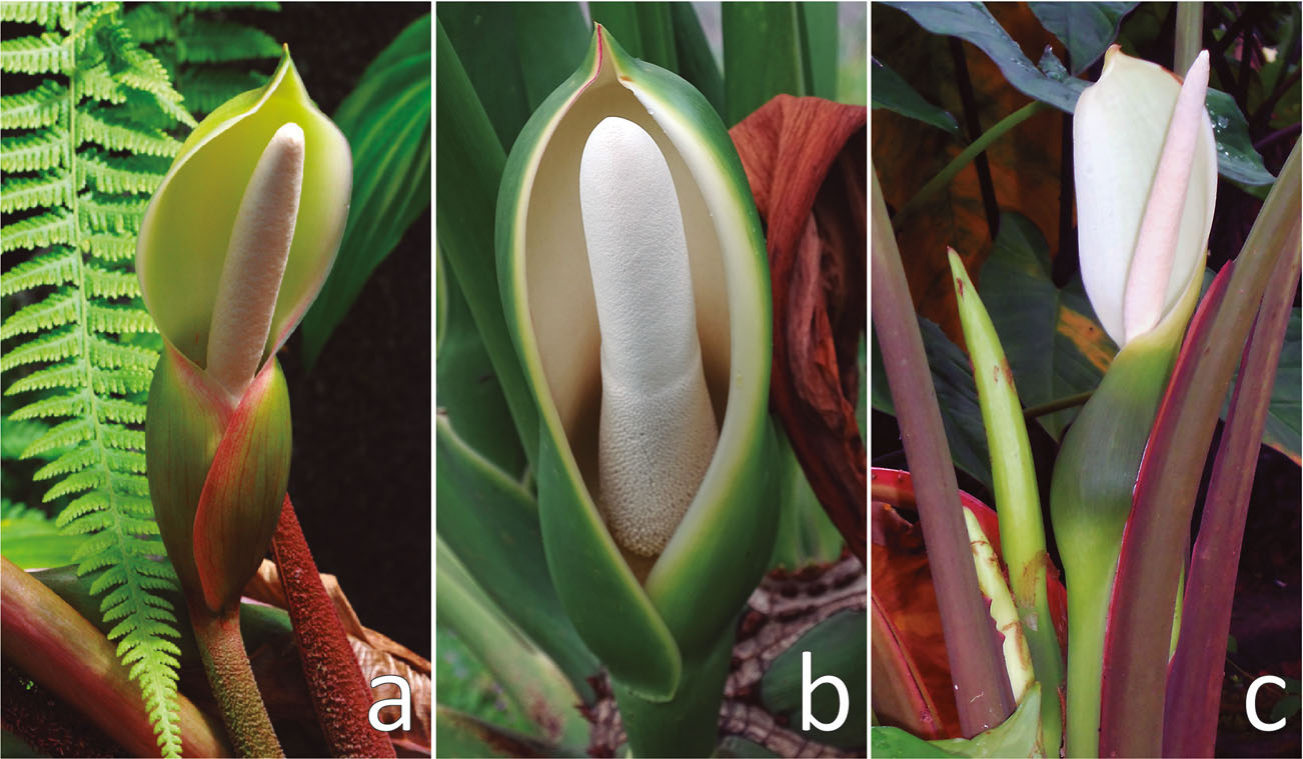
Overview of inflorescences of the three species of Araceae investigated in this study. **a**) *Philodendron squamiferum*, **b**) *Thaumatophyllum mello-barretoanum*, and **c**) *Xanthosoma hylaeae.* Authorship of (**a**) credited to David Scherberich

*Philodendron squamiferum* Poepp.: hemiepiphytic climber native to French Guiana, Suriname, and northern Brazil. Its inflorescences are funnel-shaped and composed of an approximately 10 cm long spathe, red colored in the external surface and white internally, with dark red striations within the inferior floral chamber. The white spadix, ca. 9 cm long, bears unisexual staminate and pistillate flowers. The pistillate flowers occupy the proximal portion (3–4 cm) of the spadix, whereas the staminate flowers are located on its distal portion (3–5 cm). There is also a small intermediate zone (0.6–1.2 cm) comprised of sterile staminate flowers (Fig. 2). In Northern French Guiana (Kourou region), the pollination of *P. squamiferum* has been mostly attributed to *Cyclocephala simulatrix* Höhne, 1923, but *Cyclocephala tylifera* Höhne, 1923 are also occasional floral visitors (Gibernau and Barabe 2002). The plants used for headspace sampling were grown in the living aroid collection of Universidade Católica de Brasília (HBUCB), from material originally collected in the state of Amapá, Northern Brazil (*N* = 2 individuals, *n* = 2 samples).

*Thaumatophyllum mello-barretoanum* (Burle-Marx ex G.M. Barroso) Sakur., Calazans & Mayo (Sakuragui et al. 2018): erect arborescent herb natively found in Central Brazil to Bolivia (Mayo 1991), but cultivated as an ornamental worldwide (Bown 2000). Its funnel-shaped inflorescences are composed of a 11–15 cm long spathe, succulent, dark green colored externally and creamy white internally. The white spadix exhibits pistillate flowers occupying the proximal portion (2.7–4 cm), whereas the staminate flowers are distally located (8.5–10 cm). There is a small intermediate zone (0.5–1 cm) of staminate flowers (Fig. 2). Natural populations of *T. mello-barretoanum* are associated with *Cyclocephala atricapilla* Mannerheim (Gottsberger et al. 2013). Headspace samples of *T. mello-barretoanum* were obtained from a large individual growing in the ornamental garden of the Department of Fundamental Chemistry of Universidade Federal de Pernambuco, from material originally collected at the municipality of Brasília, Brazil (*N* = 1 individual, *n* = 5 samples).

*Xanthosoma hylaea* Engl. & K. Krause: seasonally dormant geophyte, widespread in the Amazon basin, from northern Brazil to Colombia and Bolivia. When mature, its inflorescences exhibit a uniformly white spathe blade (8–12 cm long). The spathe tube (3–5 cm long) forms a distinct floral chamber, isolated from the upper portion except for a small constriction (ca. 0.5 cm diam.). Both its external and internal surfaces are evenly colored light green, with eventual deep purple coloration within the inner surface of the floral chamber. The spadix is whitish and about 1 cm shorter than the spathe, being comprised of an upper zone of fertile staminate flowers (3–7 cm long), an intermediate zone of sterile male flowers (1–2 cm long), and a lower zone of female flowers (1–2 cm female). The diameter of the spadix is about 0.6–0.8 cm in both male and female zones, but thinner (0.2–0.4 cm wide) in the length of the intermediate zone, along the constriction (Fig. 2). There is no information published on the reproductive biology of *X. hylaeae*, but it is assumed that cyclocephaline scarabs are the effective pollinators of natural populations of the species, as it is the case for other congenerics (García-Robledo et al. 2004; Milet-Pinheiro et al. 2017; Valerio 1988). The plants used for headspace sampling were grown in the living aroid collection of Universidade Católica de Brasília (HBUCB), from material originally collected in northwestern Brazil (*N* = 2 individuals, *n* = 3 samples).

### Floral Scent Collection

Floral scent samples were obtained through dynamic headspace collection (Maia et al. 2014), for which we used freshly cut inflorescences, cleanly excised at the base of the peduncle and kept in H_2_O during the intervals of highest perceivable odor emission of the female (day 1) phase of anthesis. Although we did not control the possible influence of the excision in the overall emission of floral VOCs, scent emission does not seem to be affected by manipulation and “subtle” spadix tissue damage in aroids (Skubatz et al. 1996). Cut inflorescences keep attractiveness to pollinators. Inflorescences were individually enclosed from the base of the spathe within PET film oven bags (Bratschlauch, Melitta GmbH, Germany), from which scented air was drawn for 15–45 min by a rotary vane micropump (Rietschle Thomas, Puchheim, Germany) at a constant flow rate of 150–350 mL•min^−1^. The VOCs inside the bags were trapped in glass tubes containing adsorbent material (500 mg), consisting of a 1:1 mixture of Tenax™ TA (80/100 mesh, Supelco, USA) and Carbopack™ X (40/60 mesh, Supelco, USA). Immediately following scent collection, the adsorbent traps were eluted with 250–350 μL hexane (≥99.7% purity, Sigma–Aldrich, USA). To detect environmental contaminants, negative controls were simultaneously collected from empty bags by using the same aforementioned protocol. All headspace samples were stored in 2 mL clear vials with Teflon lined caps at −24 °C for processing procedures in the laboratory.

### Chemical Characterization of Floral Bouquets by GC/MS

Headspace samples were analyzed on a gas chromatograph coupled to a mass spectrometer (GC/MS; Agilent 7890A™ gas chromatograph, Agilent 5975C Series MSD™ mass spectrometer, Palo Alto, USA), equipped with a HP-5 ms column (Agilent J&W; 30 m × 0.25 mm i.d., 0.25 μm film thickness). For each sample, 1 μL was injected in split mode (1:10–1:30) with the injector temperature set to 250 °C. The oven temperature was set at 40 °C for 2 min, then increased at a rate of 4 °C min^−1^ to 230 °C. The final temperature was held for 5 min. Helium carrier gas was maintained at a constant flow of 1.0 mL•min^−1^. MS source and quadrupole temperatures were set at 230 °C and 150 °C, respectively. Mass spectra were recorded in EI mode (70 eV) with a scanning speed of 1.0 scan•s^−1^ from *m/z* 35–450. The peak areas on the chromatograms were integrated to obtain the total ion current, which was used to determine the relative percentages of each peak. Compounds were initially identified by comparing their mass spectra and retention indices with those of authentic reference samples available from commercial mass spectral libraries (MassFinder4, NIST11 and Wiley Registry™ 9th Edition), incorporated to the software Agilent MSD Productivity ChemStation (Agilent Technologies, Palo Alto, USA). Unidentified compounds accounting to over 5% relative percentage in the samples were targeted for compound isolation and identification by further analytical processing (see below).

The three originally analyzed samples of *X. hylaeae*, which contained two unidentified compounds in relative amounts ranging from 2.0–12.1% (KI 1301 on a DB-5 equivalent column) and 37.2–91.1% (KI 1415 on a DB-5 equivalent column) (Milet-Pinheiro et al. 2017), were pooled and concentrated under a laminar N_2_ flow until apparent total solvent evaporation. Following concentration, the pooled sample was resuspended in benzene-*d*_6_ (100 atom % D, Armar Chemicals, Germany) to a total volume of 580 μL and directly used for NMR analysis. Available samples of *T. mello-barretoanum* (*n* = 5) and *P. squamiferum* (*n* = 2) were also pooled and concentrated under N_2_ to final volumes of 950 and 500 μL, respectively. These were then subjected to preparative GC for purification and fraction collection of target constituents.

### Preparative Gas Chromatography

Purification and fraction collection of the target constituents in the floral scent bouquets of *T. mello-barretoanum* and *P. squamiferum* were accomplished by preparative gas chromatography using a gas chromatograph with a flame ionization detector (Agilent 7890A, Santa Clara, USA) coupled to a preparative fraction collector (Gerstel PFC, Gerstel, Mühlheim, Germany). For the analyses with the sample of *P. squamiferum*, a low polarity ZB-5 fused silica capillary column (30 m × 0.32 mm ID, 0.25 μm, Phenomenex, Torrance, USA) was used. A high polarity ZB-Wax (30 m × 0.32 mm ID, 0.25 μm, Phenomenex, Torrance, USA) was chosen for analyses of the sample of *T. mello-barretoanum*, as it optimally separated the target compounds (see Figure S1, SI). The column effluent was split by a μFlow splitter (Gerstel, Mühlheim, Germany) into two deactivated capillary columns leading to the FID (2 m × 0.15 mm i.d.) and PFC (1 m × 0.2 mm i.d.). N_2_ makeup gas set at a flow rate of 25 mL•min^−1^ was applied to the splitter.

Aliquots of 2 μL were injected in splitless mode in a split/splitless injector (Agilent Technologies, Palo Alto, USA), which was heated to 250 °C. The temperature of the GC oven was raised from 40 °C to 200 °C at a heating rate of 20 °C min^−1^. H_2_ was used as the carrier gas with a constant flow rate of 3 mL•min^−1^. The PFC transfer line was heated to 200 °C. Modified glass tubes filled with 50 mg of Carbotrap B (mesh 20–40, Supelco, Bellefonte, USA) were used for VOC trapping. The first target floral scent compound (**1**) of *T. mello-barretoanum* was collected from 6.57 to 6.80 min (Figure S1a), for which two separate fractionated samples were obtained following 85 and 110 injection cycles. The second target floral scent compound (**2**) of *T. mello-barretoanum* was collected from 6.28 to 6.55 min, for which also two fractionated samples were acquired after 75 and 85 injection cycles, respectively. The target compound **4** in the sample of *P. squamiferum* was collected from 5.88 to 6.25 min (Figure S2b), for which two separate fractionated samples were obtained following 40 and 60 injection cycles. The glass tube traps were eluted with 400 μL dichloromethane-*d*_2_ (99.96 atom % D, Sigma-Aldrich, USA) and stored at −24 °C until NMR analysis.

### Analysis of target compounds by HR-EIMS and NMR

HR-EIMS data were recorded on an Agilent 6890 gas chromatograph (Agilent Technologies, Palo Alto, USA) equipped with a ZB-5 ms fused silica capillary column (30 m × 0.25 mm ID, 0.25 μm, Phenomenex, Torrance, USA). A split injection port at 270 °C was used for sample introduction and the split ratio was set to 10:1. The oven temperature was set at 50 °C for 5 min, then increased at a rate of 10 °C min^−1^ to 320 °C. The final temperature was held steady for 5 min. The He carrier gas was set to a constant flow rate of 1.0 mL min^−1^.The transfer line was kept at 270 °C. A JMS-T100GC (GCAccuTOF, JEOL, and Japan) Time of Flight mass spectrometer was used in electron ionization (EI) mode at 70 eV, controlled by the JEOL MassCenter™ workstation software. The source and transfer line temperatures were set at 200 °C and 270 °C, respectively. The detector voltage was set at 2000 V. The acquisition range was from *m/z* 41–600, with a spectrum recoding interval of 0.4 s. The system was tuned with PFK to achieve a resolution of 6000 (FWHM) at *m/z* 292.9824.

1D and 2D NMR experiments were performed on DRX-400 and AV II-600 instruments (Bruker) with tetramethylsilane as internal standard (TMS, δ = 0 ppm) to identify the target compounds of *T. mello-barretoanum* and *P. squamiferum*. The sample of *X. hylaea* was investigated on a 600 MHz Avance III HD Bruker spectrometer at 298 K. Chemical shift assignment was achieved with ^1^H-^1^H TOCSY, ^1^H-^13^C HSQC, ^1^H-^13^C HMBC and ^1^H spectra. Instead of a ^1^H-^1^H COSY, a TOCSY spectrum with only 12 ms mixing time was used to obtain a phase-sensitive spectrum (named COSY in the text). Chemical shifts were referenced to TMS using an external sample of 0.1% TMS dissolved in benzene-*d*_6_ (100 atom % D, Armar Chemicals, Germany). More details are found in the Supplementary Material.

## Results

### Floral Scent Composition

**Fig. 3:**
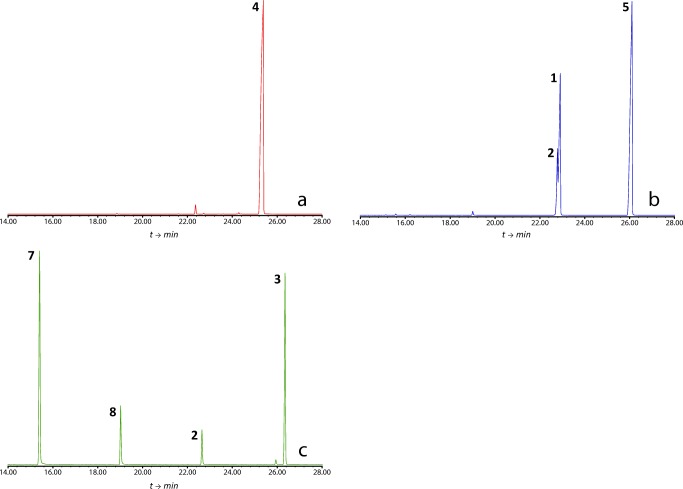
Total ion chromatograms (GC/MS obtained on a DB-5 equivalent column) of representative headspace samples of **a**) *Philodendron squamiferum*, **b**) *Thaumatophyllum mello-barretoanum*, and **c**) *Xanthosoma hylaeae.***1:** (*Z*)-4-methylene-5-(pent-2-en-1-yl)cyclopent-2-en-1-one; **2**: (*Z*)-3-methylene-2-(pent-2-en-1-yl)cyclopentan-1-ol; **3**: (*Z*)-3-methylene-2-(pent-2-en-1-yl)cyclopentyl acetate; **4**: (*E*)-4,8-dimethylnona-1,3,7-trien-5-yl acetate; **5**: (*Z*)-jasmone; **7**: methyl benzoate; **8**: methyl salicylate

The analytical investigation of the headspace floral scent samples of *P. squamiferum*, *T. mello-barretoanum* and *X. hylaea* by GC/MS is summarized in Table 1. Representative gas chromatograms are shown in Fig. 3. The bouquet of *T. mello-barretoanum* is dominated by (*Z*)-jasmone (**5**) and also contains the novel compounds **1** and **2** as major constituents. In contrast, samples of *P. squamiferum* lack any jasmone derivatives and instead present as a dominant constituent a novel homoterpene acetate (**4**). In samples of *Xanthosoma hylaea,* the major constituent is yet again a jasmone derivative, acetate **3**, together with methyl benzoate (**7**) and methyl salicylate (**8**), in varying relative abundances. The related alcohol **2** is also present in minor amounts. All other constituents are known and were identified, except for small amounts of a compound with an identical mass spectrum to that of acetate **4**, evidenced at an earlier retention time in a low polarity phase (KI 1386 on a DB-5 equivalent column). We therefore tentatively identified this compound as the (*Z*)-diastereomer of compound **4** (Table 1).

**Table 1:** Chemical composition (min-max relative amounts of each compound) of the floral scents of *Philodendron squamiferum*, *Thaumatophyllum mello-barretoanum* and *Xanthosoma hylaeae* (Araceae)

	RI	*P. squamiferum* (n = 1)	*T. mello-barretoanum* (n = 3)	*X. hylaea* (n = 3)
Total number of compounds		3	10	5
Benzenoids and phenylpropanoids				
Methyl benzoate (**7**)	1095	–	–	4.59–44.27
Methyl salicylate (**8**)	1194	0.17	0.49–1.22	2.06–11.77
Jasmone derivates				
(*Z*)-3-Methylene-2-(pent-2-en-1-yl)cyclopentan-1-ol (**2**)	1301	–	10.76–16.16	2.00–12.08
(*Z*)-4-Methylene-5-(pent-2-en-1-yl)cyclopent-2-en-1-one (**1**)	1305	–	15.96–27.99	–
(*Z*)-Jasmone (5)	1407	–	60.32–60.66	0.06–2.94
(*Z*)-3-Methylene-2-(pent-2-en-1-yl)cyclopentyl acetate (**3**)	1415	–	–	37.17–91.10
Monoterpenes				
β-Myrcene	991	–	0.1	–
(*Z*)-β-Ocimene	1039	–	0.12	–
(*E*)-β-Ocimene	1049	–	0.12–3.35	–
α-Terpinolene	1088	–	0.04–1.12	–
Linalool	1100	–	0.14–0.40	–
Homoterpenes		–		–
(*E*)-4,8-Dimethyl-1,3,7-nonatriene (**6**)	1117	0.17	0.07–0.14	–
(*Z*)-4,8-Dimethylnona-1,3,7-trien-5-yl acetate	1295	1.62	–	–
(*E*)-4,8-Dimethylnona-1,3,7-trien-5-yl acetate (**4**)	1386	97.7	–	–

Floral scent samples were obtained by dynamic headspace sampling during the interval of highest perceivable odor emission in the course of the female phase (day 1) of anthesis. The compounds are listed according to compound class and gas chromatographic retention index (RI; HP–5 ms phase). Compounds were only included if they accounted for ≧ 0.1% of the total amount in any sample

### Structural Elucidation of the Compounds **1–4**

**Fig. 4:**
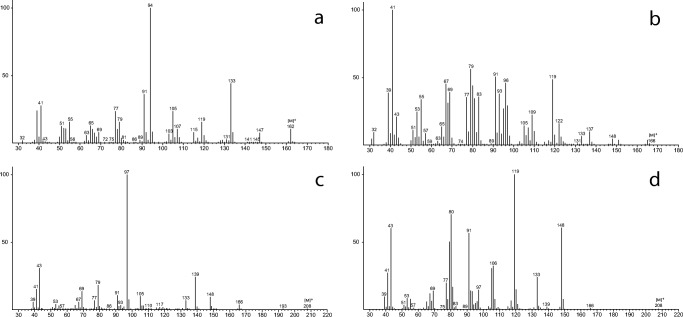
EI mass spectra of four major compounds of floral headspace scent collections from *Philodendron squamiferum*, *Thaumatophyllum mello-barretoanum* and *Xanthosoma hylaeae.* The compounds were identified in this study as **a**: (*Z*)-4-methylene-5-(pent-2-en-1-yl)cyclopent-2-en-1-one (**1**); **b**: (*Z*)-3-methylene-2-(pent-2-en-1-yl)cyclopentan-1-ol (**2**); **c**: (*Z*)-3-methylene-2-(pent-2-en-1-yl)cyclopentyl acetate (**3**); **d**: (*E*)-4,8-dimethylnona-1,3,7-trien-5-yl acetate (**4**)

The GC/MS analyses of headspace floral scent samples of *P. squamiferum*, *T. mello-barretoanum* and *X. hylaea* revealed four major constituents with unknown mass spectra (Figs. 3 and 4).

The molecular formula of compound **1** was determined by HRMS to be C_11_H_14_O (obs. *m/z* 162.10475, calcd. *m/z* 162.10446), indicating the presence of five double-bond equivalents. The ^1^H-NMR data are shown in Table 2. ^13^C-NMR and DEPT data revealed one carbonyl group at δ_C_ 208.6 and six olefinic carbons between δ_C_ 113.1 and 159.2 (Table 3), suggesting one ring in the molecule. The olefinic carbons consisted of one quaternary carbon, four methines and a terminal methylene group (C-6). The methine signals at δ_H_ 7.74 (H-4) and 6.22 (H-5) are typical for an α,β-unsaturated carbonyl system. The allylic ^4^*J-*coupling between the terminal H-6 protons to H-4 and H-2 indicated an exo-methylene group and an α,β,γ,δ-conjugated carbonyl system. Additional ^1^H,^1^H-COSY and HMBC data (Figure S2, SI) established a 2-pentenyl side chain located next to the carbonyl group of a cyclopentenone ring. Including the allylic system, only a single spin system was evident from the ^1^H,^1^H-COSY experiment. The double-bond at C-8/C-9 was determined to be *cis*-configured by the NOESY coupling of H-8 to H-9. The data are consistent with the structure (*Z*)-4-methylene-5-(pent-2-en-1-yl)cyclopent-2-en-1-one for compound **1**.

**Table 2:** ^1^H-NMR Data for compounds **1**–**4** (δ in ppm, *J* in Hz)

Atom	1	2	3^a^	4
1	–	3.98, dd (5.8)	5.05, dd (9.9, 4.9)	5.12, dd (10.2, 2.0) 5.22, dd (16.8, 2.0)
2	2.85–2.82, m	2.30–2.26, m	2.59, m	6.57, ddd (16.8, 10.9, 10.2)
3	–	–	–	6.03, dq (10.9, 0.7)
4	7.74, d (5.6)	2.51–2.24, m	2.36, m^b^ 2.14, m^b^	–
5	6.22, dq (5.6, 0.8)	1.97–1.56, m	1.89, m 1.65^b^	5.09, t (6.8)
6	α: 5.40–5.39, m β: 5.34–5.33, m	4.91–4.86, m	4.93, m^b^	2.41–2.27, m
7	2.55–2.46, m	2.29–2.15, m	2.23, (t)^b^ 2.15, m^b^	5.03, tq (7.2, 1.4)
8	5.24–5.19, m	5.51–5.42, m	5.47^b^	–
9	5.43-5.38, m	5.51–5.42, m	5.47 ^b^	1.62, s
10	2.06–2.00, m	2.08, m	1.99 ^b^	1.75, s
11	0.92, t (7.5)	0.96, t (7.5)	0.92 ^b^	1.69, s
12	–	–	–	–
13	–	–	1.65 ^b^	2.01, s

^a^in C_6_D_6_ as solvent

^b^signals not isolated in 1D

**Table 3:** ^13^C-NMR Data for compounds 1–4 (δ in ppm)

Atom	1	2	3^a^	4
1	208.6	77.7	79.4	117.8
2	48.0	52.8	49.8	132.8
3	149.3	153.8	152.1	127.7
4	159.2	29.7	30.1	136.7
5	134.30	33.0	30.1	78.8
6	113.1	106.5	106.8	32.0
7	27.4	30.1	30.2	119.3
8	124.5	127.3	126.1	134.7
9	134.32	133.3	133.0	18.0
10	21.0	21.0	20.7	12.9
11	14.3	14.4	14.1	25.8
12	–	–	169.4	170.4
13	–	–	20.5	21.3

^a^in C_6_D_6_ as solvent

The molecular formula of compound **2** was determined by the HREIMS [M-H]^+^ ion at *m/z* 165.12764 (calcd 165.12794) to be C_11_H_18_O, indicating three double bond equivalents. The [M-H]^+^ ion was more abundant than the [M]^+^ ion, a pattern often found in alcohols. A terminal exo-methylene group was indicated again by the ^13^C and DEPT data for C-6 (δ_C_ 106.5) and the quaternary C-3 (δ_C_ 153.8). The chemical shift of C-1 (δ_C_ 77.7) showed that the carbonyl group of **1** is reduced to an alcohol in **2**. ^1^H,^1^H-COSY, HSQC and HMBC confirmed again a 2-pentenyl side chain (Figure S2). ^1^H,^1^H-COSY cross peaks of H-2 to H-1, H-1 to H-5 and H-5 to H-4 as well as HMBC cross peaks of H-1 to C-3 and C-4 revealed the 1,2,3-trisubstituted cyclopentyl-ring, also occurring in **1**. Obviously not only the carbonyl group was reduced compared to **1**, but also the internal ring double-bond. In summary, compound **2** is (*Z*)-3-methylene-2-(pent-2-en-1-yl)cyclopentan-1-ol. Whether the substituents of the cycopentanol-ring are *cis*- or *trans*-configured could not be revealed conclusively by the NMR data because of the flexibility of the ring system.

The NMR data of compound **3** were in close agreement with those found for compound **2** (Tables 2 and 3), but showed two additional ^13^C signals. Both ring and side chain signals closely matched, and the different 2D NMR experiments delivered similar results for both compounds (Figures S3-S5 SI). The largest difference was found for the ^1^H-NMR signal of H-1 that shifted to 5.05 ppm, indicating an ester carbinol. The ^13^C data of C-12 (δ_C_ 169.4) and C-13 (δ_C_ 20.4) are typical for an acetyl group. This group was connected to C-1, visible by a weak HMBC correlation. Therefore, compound **3** was identified as (*Z*)-3-methylene-2-(pent-2-en-1-yl)cyclopentyl acetate. Interestingly, the mass spectra of **2** and **3** differed largely (Fig. 4). This was unexpected due to the fact that often alcohols and their acetates share many features in their mass spectra.

The molecular formula of compound **4** was determined by HR-EIMS to be C_13_H_20_O_2_ (obs*. m/z* 208.14722, calcd *m/z* 208.14633), indicating four double bond equivalents. The ^1^H-NMR showed six signals in the olefinic region (δ_H_ 6.57, 6.03, 5.22, 5.12, 5.09 and 5.03) as well as five aliphatic signals (δ_H_ 2.41–2.27, 2.01, 1.75, 1.69 and 1.62), four of which were methyl singlets. ^13^C-NMR and DEPT spectra showed 13 signals.

Furthermore six olefinic carbons were present, consisting of two quaternary carbons, three methines and one methylene. The five aliphatic carbons consisted of three methyl groups, one methine and one CH_2_-group. The presence of an acetate moiety was indicated by the signals at δ_C_ 170.4 and 21.3 and δ_H_ 2.01. ^1^H,^1^H-COSY analysis revealed three spin systems (shown in bold in Figure S2, SI). The isohexene system was elucidated by HMBC couplings of H-9 to C-11 and C-7, H-11 to C-9 and C-7, the COSY coupling of H-7 to H-6 and the HMBC couplings of H-6 to C-8. Carbinol C-5 was connected to this spin system by HMBC coupling of H-5 to C-7 and COSY coupling of H-6 to H-5. The second pentadiene spin system was elucidated starting from the methylene end. ^1^H-NMR and COSY experiments revealed a *trans*-coupling for H-1_β_ to H-2 (16.8 Hz) and a *cis*-coupling of H-1_α_ to H-2 (10.2 Hz), confirming the terminal double bond. The system was extended by coupling of H-2 to H-3 (10.9 Hz), followed by allylic ^4^*J-*coupling of H-3 to H-10 (0.7 Hz). Corresponding HMBC couplings were observed. Both spin systems were connected via HMBC couplings of H-5 to C-3, C-7 and C-10. Carbinol C-5 was therefore also connected to the third spin system, the acetyl group. The configuration of the double bond at C-3 was determined by NOE experiment to be *trans*-configured. Key NOESY couplings are H-5/H-3, H-3/H-1_β_ and H-2/H-1_α_, are shown in Figure S2 (SM). In summary, compound **4** is (*E*)-4,8-dimethylnona-1,3,7-trien-5-yl acetate.

The absolute configurations of all compounds, as well as the relative configurations of compounds **2 **and **3**, still remain to be elucidated.

## Discussion

Compounds **1**, **2** and **3** are closely related to (*Z*)-jasmone (**5**), a widely distributed floral scent component (Knudsen et al. 2006), derived from the plant hormone jasmonic acid (Koch et al. 1997; Schaller et al. 2004). High emissions of (*Z*)-jasmone have been particularly well documented among different genera of thermogenic night-blooming aroids [*Thaumatophyllum* (Gottsberger et al. 2013); *Montrichardia* (Gibernau et al. 2003); *Dieffenbachia* (Etl et al. 2016)], and this VOC is a known attractant of anthophilous insects associated with these species. Compound **1**, for which we propose the name dehydrojasmone, is an oxidized version of jasmone. Formal dehydrogenation of the cyclpentenyl-ring of jasmone would lead to the chemically very unstable cyclopentadienone ring system. Such a ring system is chemically unfavorable, because a partly antiaromatic system is formed. Therefore, the C-2—C-3-double bond has to go out of ring, but still in conjugation, which in the case of **1** leads to its exo-double-bond.

Similarly, reduction of jasmone to its alcohol would lead to an allylic alcohol prone to acid catalyzed reaction. This is yet again avoided by the exo-double bond in the alcohol **2** and its acetate** 3**, for which we propose the names isojasmol and isojasmyl acetate, respectively. Following the elucidation of its structure, we noticed that isojasmol had been previously identified by R. Kaiser as a dominant constituent in combination with (*Z*)-jasmone in heaspace floral scent samples of *Ludovia lancifolia* Brongn., an epiphytic Cyclanthaceae which grows natively in humid Neotropical forests and is pollinated by small-sized acalyptine flower weevils (Curculionidae, Acalyptini) (Teichert et al. 2018). Nevertheless, no spectroscopic data were reported.

Compound **4** is an allylic oxidation product of the homoterpene 4,8-dimethylnona-1,3,7-triene (DMNT) (**6**), a common herbivory-induced VOC (Turlings and Tumlinson 1992). Its presence in high concentrations as a floral scent is most remarkable in brood pollination systems involving *Yucca* spp. (Agavaceae) and yucca moths (Lepidoptera, Prodoxidae) (Gäbler et al. 1991; Svensson et al. 2005), but it has also been documented as a major constituent in scarab beetle-pollinated aroids of the genus *Homalomena* in Borneo (Hoe et al. 2016). Interestingly, (*E*)-cyclanthone and other unique derivatives of DMNT have been described from the floral scent of *Cyclanthus bipartitus* Poit. ex A.Rich. (Cyclanthaceae) (Schultz et al. 1999), whose inflorescences are also frequently visited by anthophilous cyclocephaline scarabs (Beach 1982).

The unique structure of these floral VOCs and their biosynthesis in large amounts imply selective evolutionary pressure exerted by scent-oriented pollinators (Raguso 2008; Schiestl and Johnson 2013). Contrastingly to more widespread floral scent constituents, such as **5**, **6**, and **7**, they might function as specific communication cues to lure certain species of cyclocephaline scarabs, an assumption supported by the fact that other rare or uncommon floral VOCs identified in the perfumes of Neotropical night-blooming Araceae proved to be attractants of these anthophilous insects (Dötterl et al. 2012; Gottsberger et al. 2012; Maia et al. 2012, 2013, 2018; Pereira et al. 2014). In a study conducted in the Atlantic Forest of southeastern Brazil, Gottsberger et al. (2013) showed that synchronous flowering populations of three different species of *Thaumathophylum* within a ca. 250 km radius, including *T. mello-barretoanum*, were each associated with an exclusive species of pollinating cyclocephaline scarab, whereas also presenting distinct qualitative floral scent compositions. A similar trend is observed in natural populations of *P. squamiferum* in northern French Guiana, whose reproductive success rely on two species of *Cyclocephala* that were only very rarely recovered from inflorescences of other syntopic and co-flowering nocturnal aroids (Gibernau 2015; Gibernau et al. 1999, 2000, 2003).

What is noteworthy is the fact that the novel compounds identified in present study all appear to be actually derived from more common precursors through biosynthetic “post-processing”, thus yielding chemical diversity as a result of enzyme-mediated hydroxylation, esterification, or oxidation (Raguso 2008). Such chemical diversification contributed to the high overall diversity of floral VOCs derived from several compound classes detected in cyclocephaline scarab-pollinated angiosperms. Future studies should test whether this high chemical diversity is paired by the variety of olfactory receptors in these specialized pollinating beetles, and whether receptors for uncommon compounds, such as the ones described in our study, are likewise derived from receptors for their most common precursors. Selective attraction of anthophilous cyclocephaline scarabs would further support the hypothesis that they are specific attractants.

## Electronic supplementary material

ESM 1 (DOCX 1950 kb)
