# Supplementary material for: Novel Floral Scent Compounds from Night-Blooming Araceae Pollinated by Cyclocephaline Scarabs (Melolonthidae, Cyclocephalini)
Source: J Chem Ecol. 2018 Sep 19;45(2):204–13. doi: 10.1007/s10886-018-1018-1 (PMC6469606; doi:10.1007/s10886-018-1018-1)
Supplement: Supplementary file 1 — (DOCX 1950 kb) [file 10886_2018_1018_MOESM1_ESM.docx]

**SuppLementary MATERIAL**

**Journal of Chemical Ecology**

Novel floral scent compounds from night-blooming Araceae pollinated by cyclocephaline scarabs

(Melolonthidae, Cyclocephalini)

Artur Campos D Maia, Christopher Grimm, Mario Schubert, Florian Etl, Eduardo Gomes Gonçalves, Daniela Maria do Amaral Ferraz Navarro, Stefan Schulz, Stefan Dötterl

^*^ Corresponding authors

Stefan Schulz

stefan.schulz@tu-bs.de

http://orcid.org/0000-0002-4810-324X

Stefan Dötterl

stefan.doetterl@sbg.ac.at

http://orcid.org/0000-0001-5228-1332

Contents

1. Preparative gas chromatography 2

2. NMR assignments of isolated compounds **1**, **2**, and **4** 3

3. NMR analysis and spectra of compound **3** 4

4. NMR spectra of compounds **1**, **2**, and **4** 8

**1. Preparative gas chromatography**


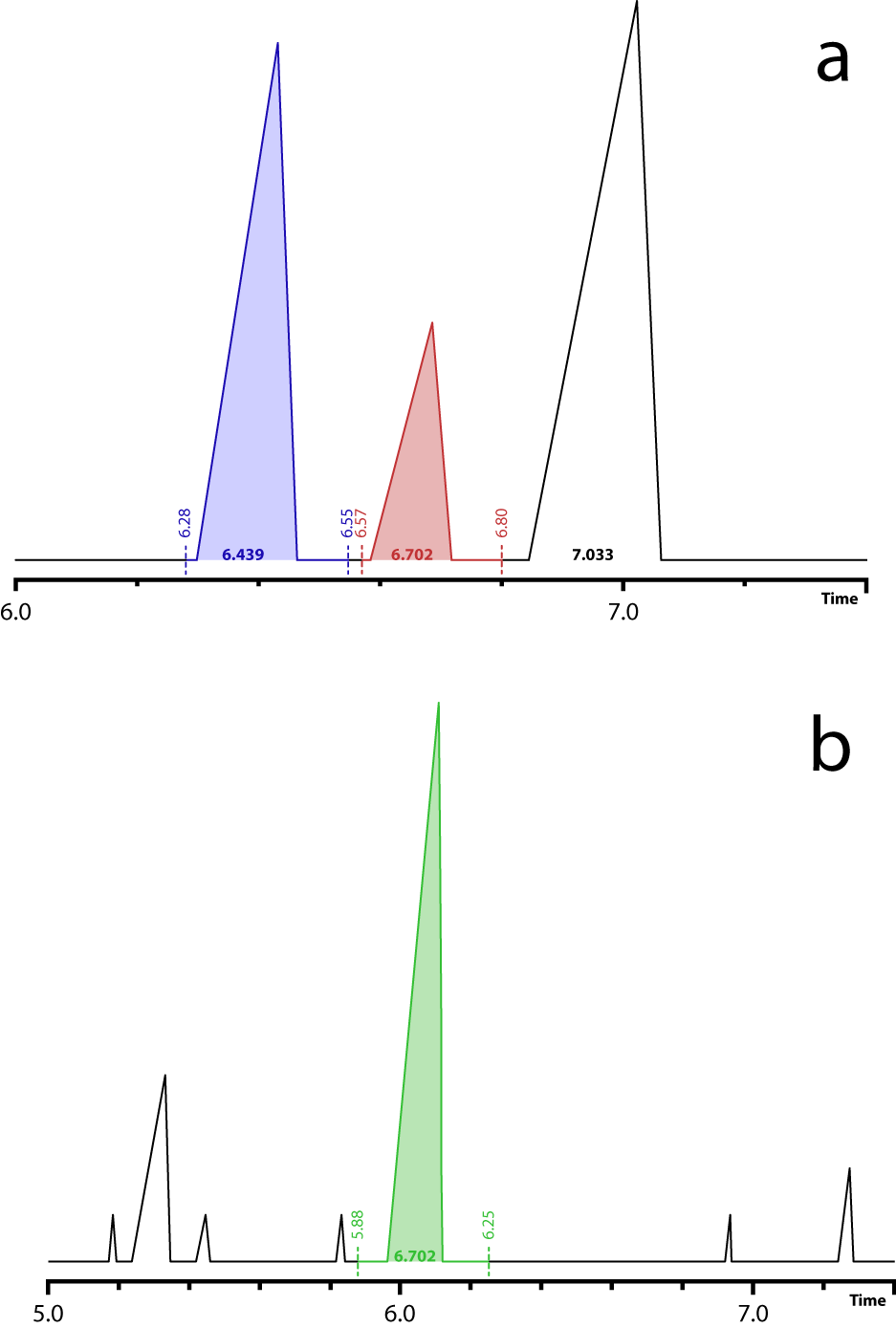


**Figure S1.** Total ion chromatograms of preparative separation of compounds a) **1** (red) and **2** (blue) on a 30m ZB-Wax phase, b) **4** (green) on a 30m ZB-5 phase.

**2. NMR assignments of isolated compounds 1, 2, and 4**

 **Figure S2.** 2D NMR experiments of compounds **1**, **2**, and **4**. ^1^H,^1^H COSY (bold bond) and HMBC correlations (blue arrows). NOE correlations are shown in the box.

**3. NMR** analysis and spectra **of compound 3**

Compound **3** was identified as mixture with other components without knowledge on its relation to compound **2**. Chemical shift assignment was achieved with ^1^H-^1^H TOCSY (total correlated spectroscopy, mixing times of 120 ms), ^1^H-^13^C HSQC (heteronuclear single quantum correlation), ^1^H-^13^C HMBC (heteronuclear multiple-bond correlation) and ^1^H spectra, using standard pulse sequences either from the Bruker library (hsqcedetgpsisp2.2, hmbcgpndqf) or from ETH Zurich. Instead of a ^1^H-^1^H COSY (correlated spectroscopy) a TOCSY spectrum with only 12 ms mixing time was used to obtain a phase-sensitive spectrum (named COSY in the text). Typical parameters were for the TOCSY 4 number of scans, 4096×512 points and 1.8 s recycle delay; for the HSQC 32 number of scans, 2048×256 points and 2 s recycle delay; for the HMBC 32 number of scans, 4096×256 points and 2 s recycle delay. Spectra were processed using Topspin 3.2 (Bruker Biospin) and analyzed using Sparky version 3.111 (T. D. Goddard and D. G. Kneller, SPARKY 3, University of California, San Francisco).

Examination of the NMR signals in a two-dimensional ^1^H-^13^C HSQC spectrum (Figure S3) revealed three olefinic signals: two CH signals at 126.1 ppm/5.47 ppm and 133.0 ppm/5.47 ppm and one CH_2_ group at 106.8 ppm/4.93 ppm. The latter methylene group (labeled as 11) served as a starting point from which seven correlated ^1^H resonances were observed in a ^1^H-^1^H TOCSY spectrum and three in a ^1^H-^1^H COSY spectrum Figure S4). The COSY correlations indicate that the H_2_C=C group is flanked by two aliphatic groups, a CH and a CH_2_ group (C5 and C3). This is confirmed by the ^1^H-^13^C HMBC spectrum (Figure S5) showing long-range correlations from H11 to both flanking carbons (C3 and C5). The four additional ^1^H correlations in the ^1^H-^1^H TOCSY spectrum originate from two methylene groups (C2 and C6). An almost identical TOCSY correlation pattern is seen for the ^1^H resonance at 5.05 ppm that originates from a CH group with a ^13^C chemical shift of 79.4 ppm. Additional COSY correlations from H1 to H2a/H2b, from H2a/H2b to H3a/H3b and from H1 to H5 support a five-membered ring (C1 to C5) with three branching sites: the olefinic CH_2_ group (C11) attached at C4, and two other extensions at C1 and C5. The extension at C5 could be established by COSY, TOCSY and HMBC correlations from the ring a methylene group (C6) and two olefinic CH-groups (C7 and C8). This extension is terminated by an ethyl group (C9 and C10), which shows many correlations to the other nuclei of the extension.

Elucidating the nature of the extension at C1 was more difficult. Its ^13^C chemical shift of 79.4 ppm suggested an oxygen as connected atom. The corresponding proton displayed with 5.05 ppm an unusual down-field shift, which could not be explained by most OR extensions. A -O-C=O- ester group could place a carbonyl sterically close to H1, which would explain the down-field shift. Interestingly the HMBC spectrum showed a strong correlation between a so far unassigned methyl group to a carbonyl signal at 169.4 ppm, to which a weak signal from H1 is visible too. These two correlations establish an acetyl-ester as extension at C1.


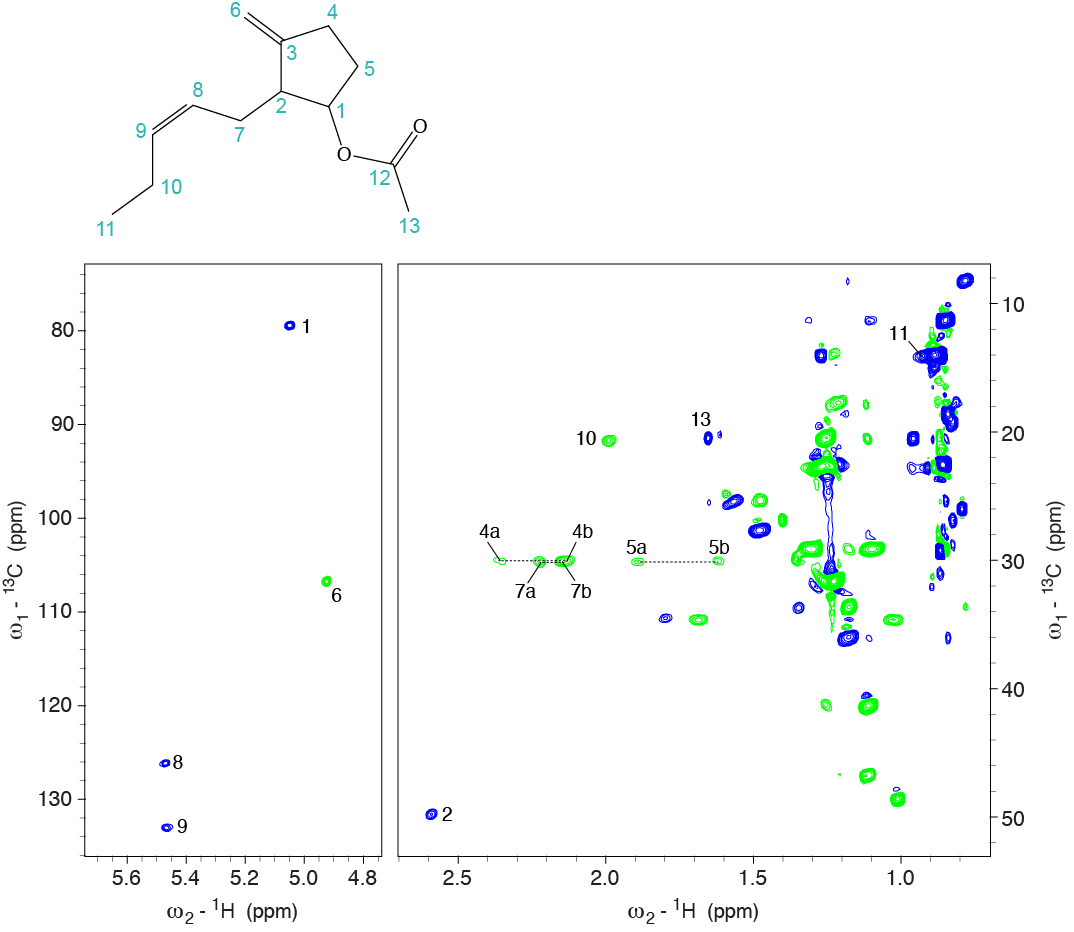


**Figure S3.** Hydrogen-carbon one-bond correlations observed in a 2D ^1^H-^13^C HSQC spectrum recorded of the head-space raw eluate of *X. hylaea* (compound **3** as main component) in benzene-*d_6_* at 298K and 600 MHz. The spectrum is ^13^C edited, so that CH_2_ groups show negative signals (green) in contrast to CH and CH_3_-groups that are positive (blue). Two regions of interest are displayed. Most signals of the compound of interest (labeled with numbers) are well separated from other aliphatic signals originating from the other components of the mixture. On the top the carbon skeleton of the compound is shown with the atom numbering.


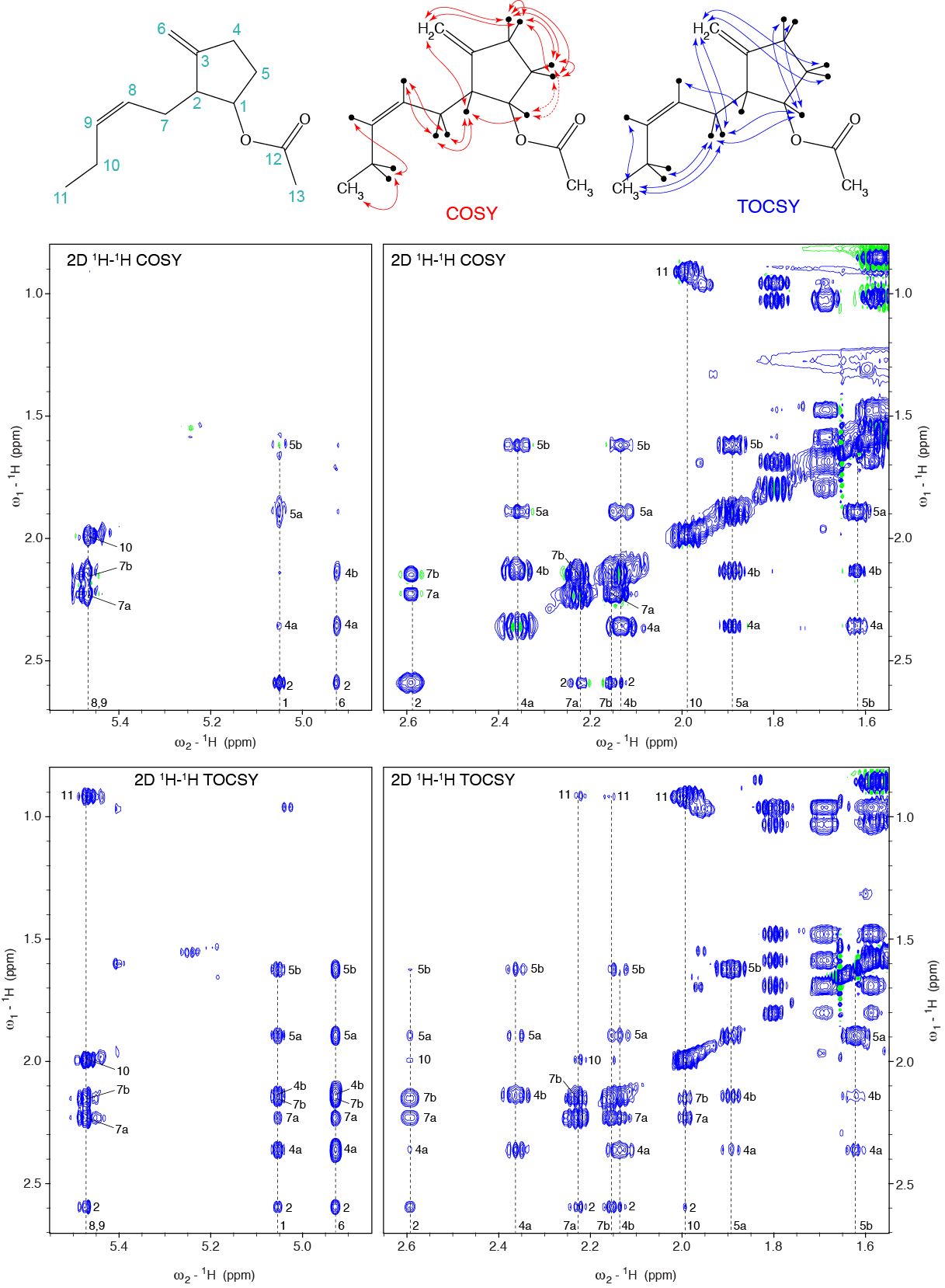


**Figure S4.** ^1^H-^1^H correlations observed in 2D ^1^H-^1^H COSY and TOCSY spectra recorded of the head-space raw eluate of *X. hylaea* (compound **3** as main component) in benzene-*d_6_* at 298K and 600 MHz. At the top the chemical structure of the compound is shown with the atom numbering (left) and the observed key correlations seen in the COSY and TOCSY spectra indicated as red arrows. In the middle, two relevant regions of the 2D ^1^H-^1^H COSY are shown with assignments, at the bottom corresponding regions of the 2D ^1^H-^1^H TOCSY.


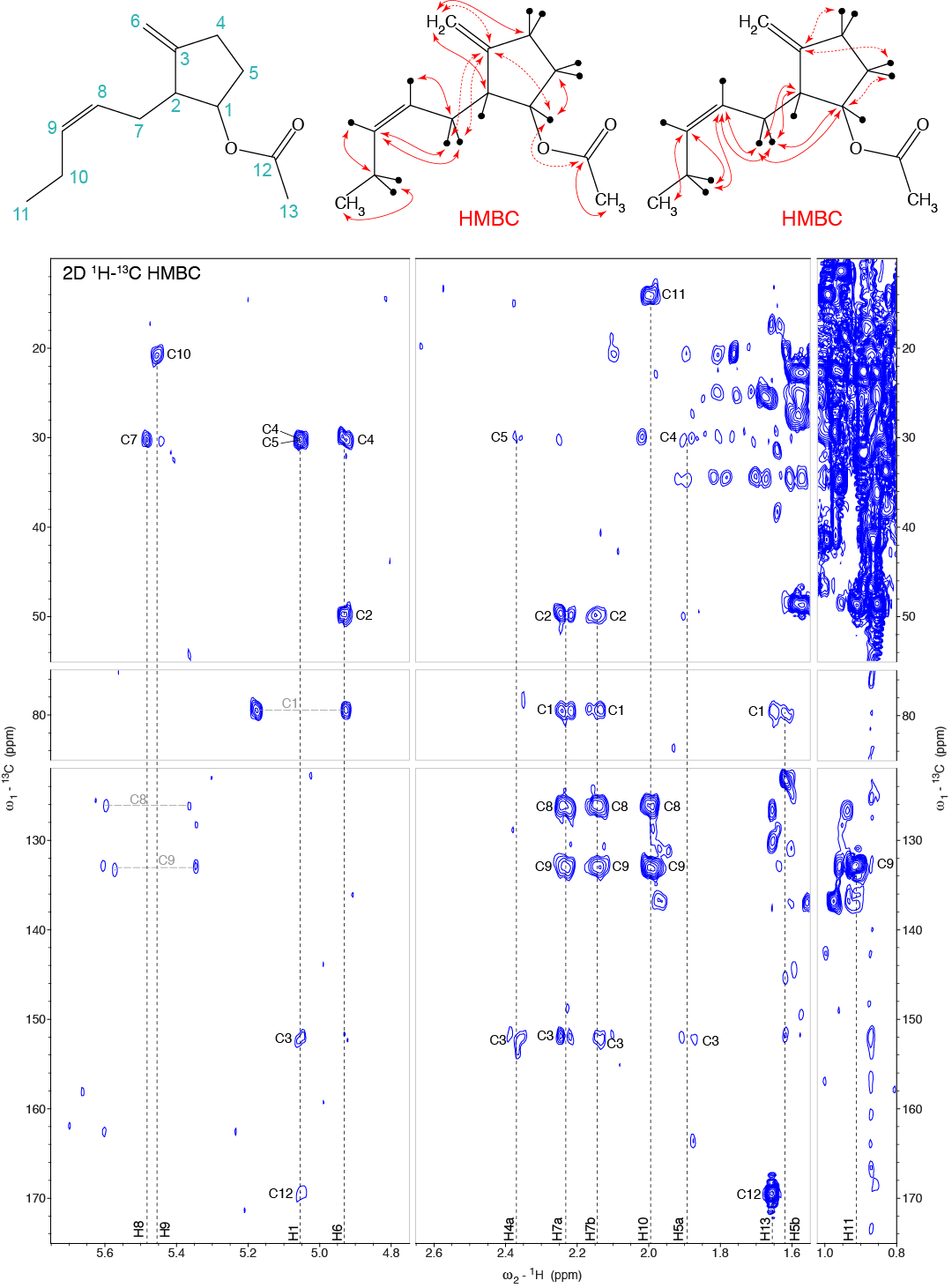


**Figure S5.** Long-range hydrogen-carbon correlations observed in a 2D ^1^H-^13^C HMBC spectrum recorded of the head-space raw eluate of *X. hylaea* (compound **3** as main component) in benzene-*d_6_* at 298K and 600 MHz. The chemical structure of the compound is shown three times at the top, with the atom numbering (left) and the observed key correlations seen in the HMBC spectrum. For clarity the large number of correlations indicated as red arrows on two structures. Nine regions of the HMBC spectrum are shown, relevant signals are labeled in black. Signals of not completely suppressed one-bond ^1^H-^13^C correlations (splitted by ^1^J_CH_) are indicated in grey.

**4. NMR spectra of compounds 1, 2, and 4**


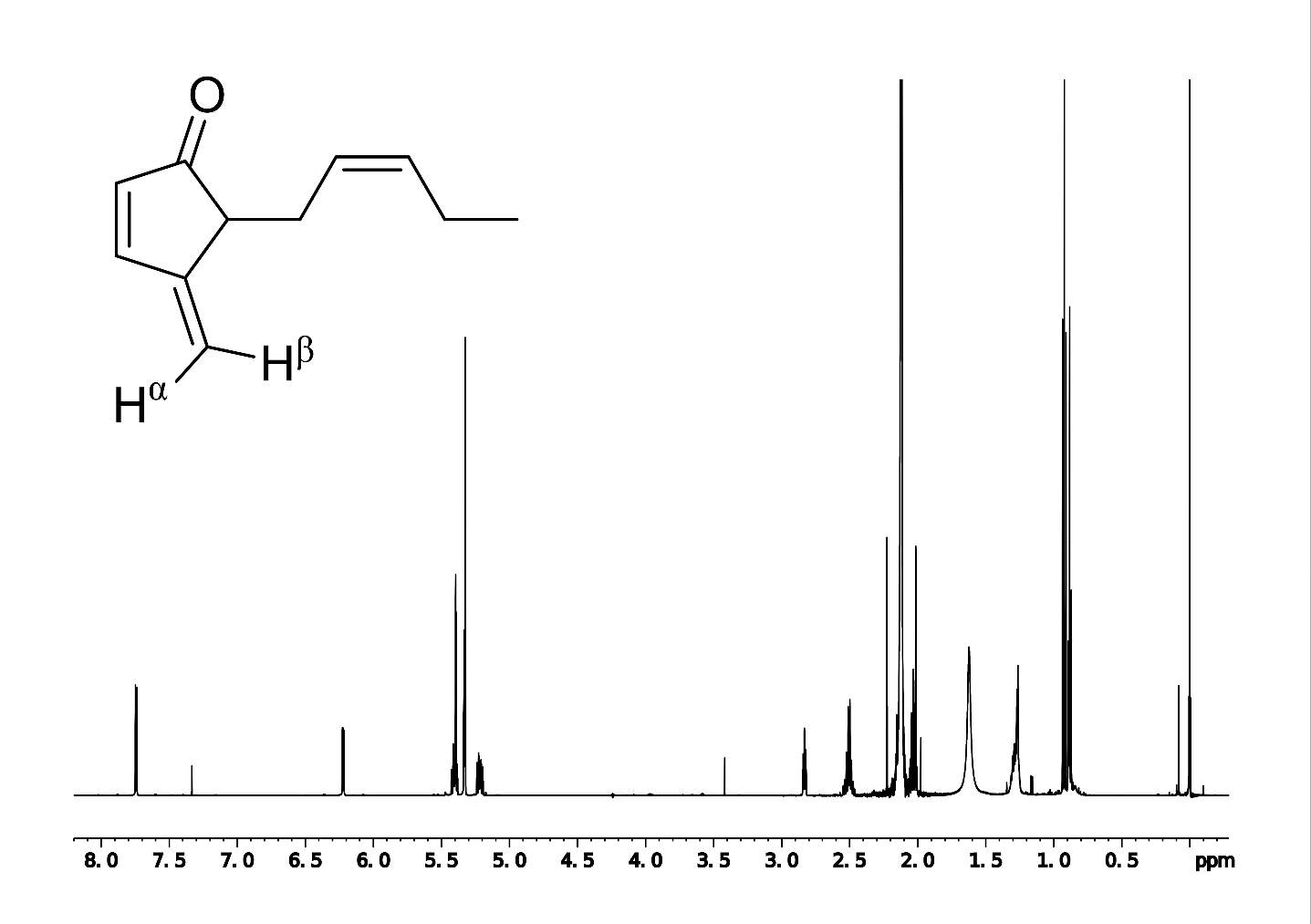
**Figure S6.** ^1^H NMR of compound **1**.


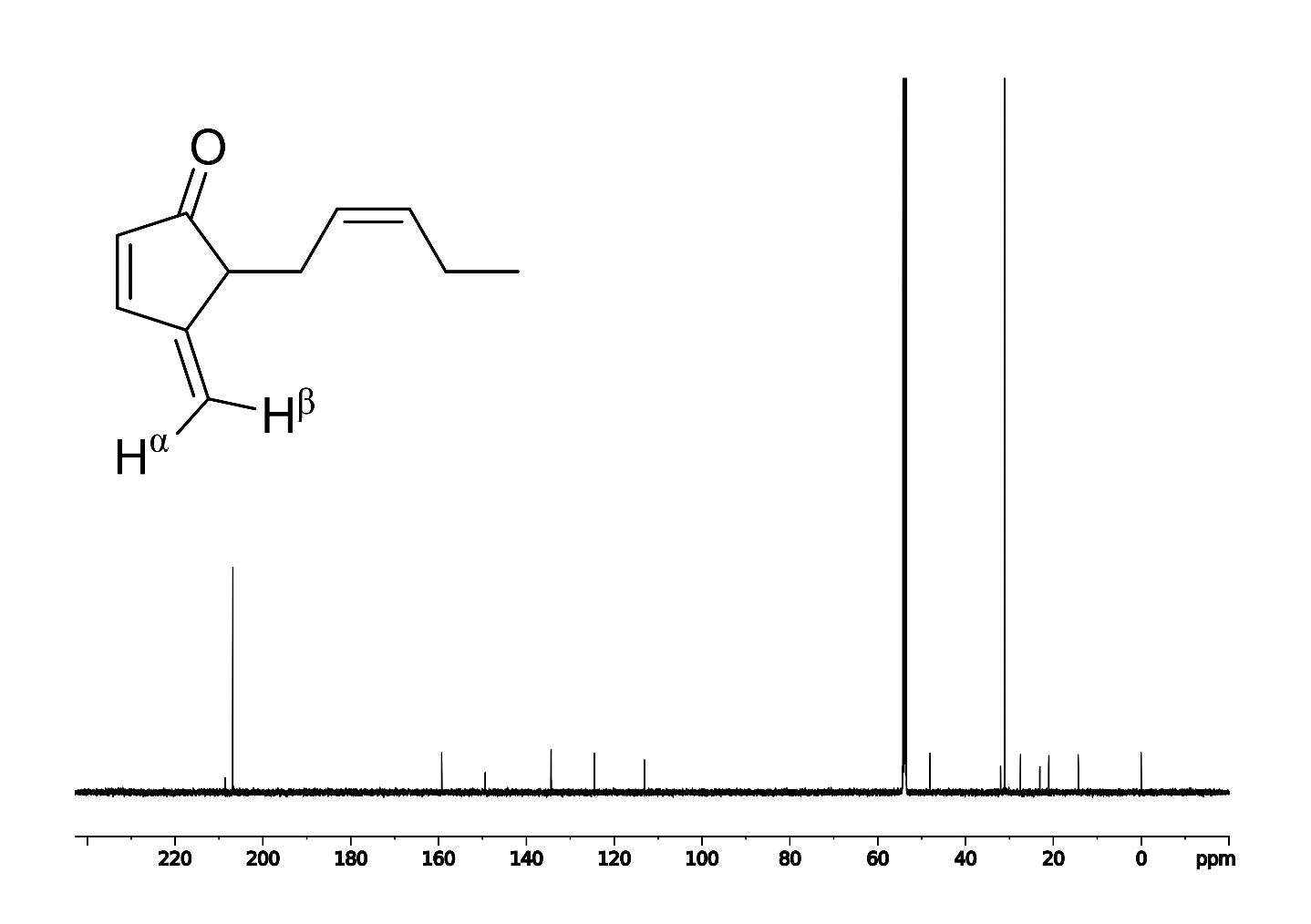
**Figure S7.** ^13^C NMR of compound **1**.


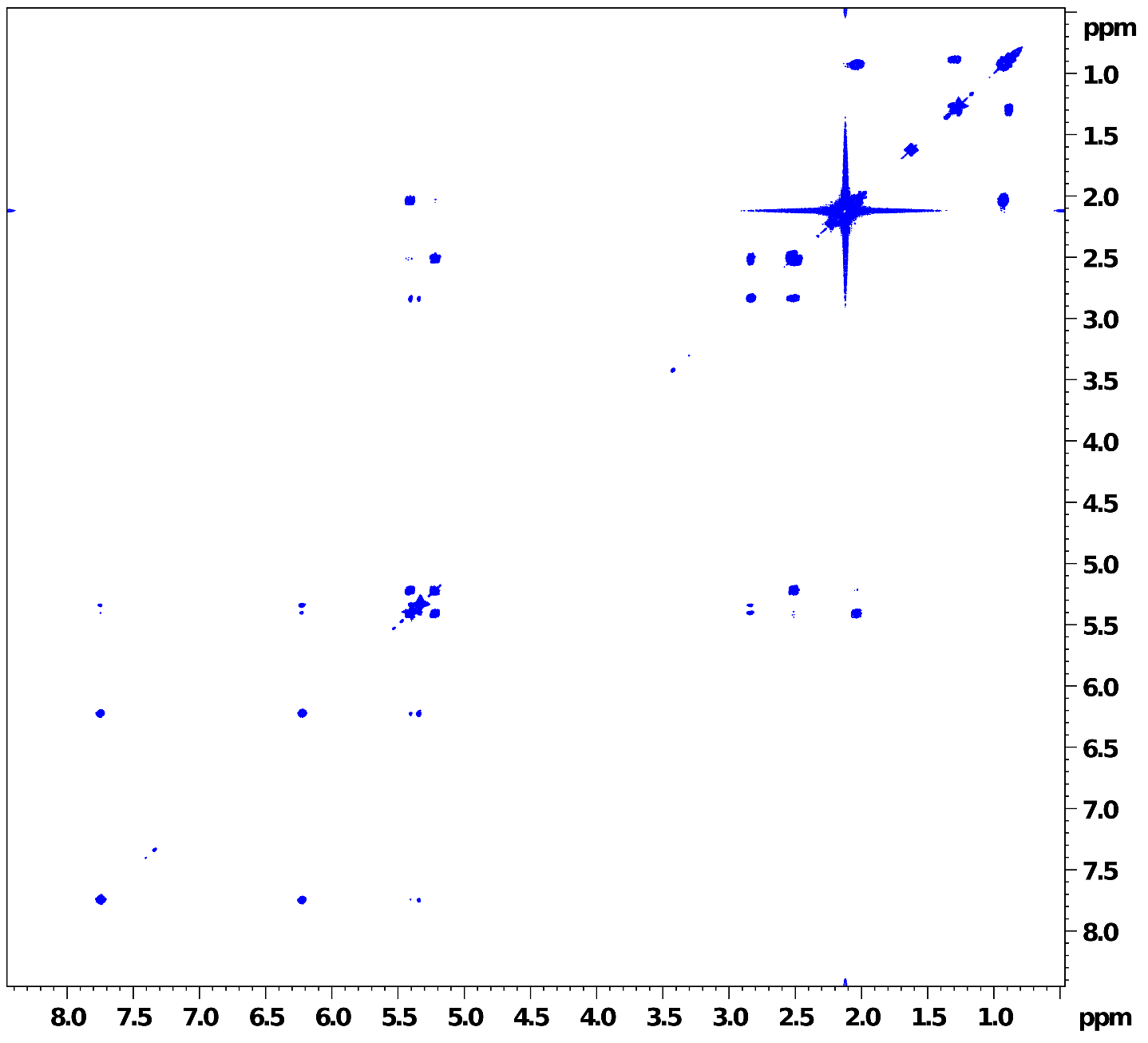


**Figure S8.** ^1^H,^1^H COSY of compound **1**.


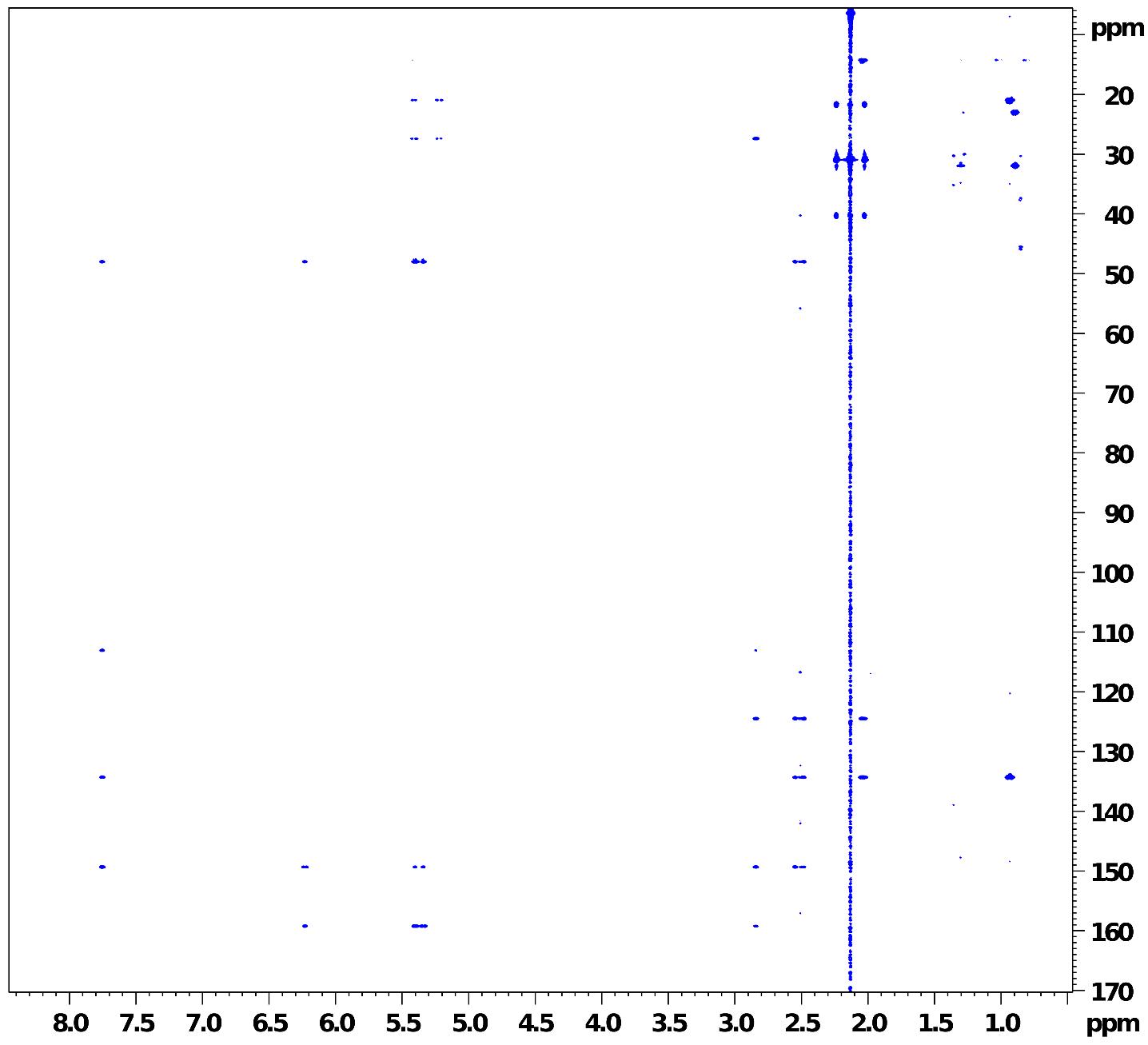


**Figure S9.** ^1^H,^13^C HMBC of compound **1**.


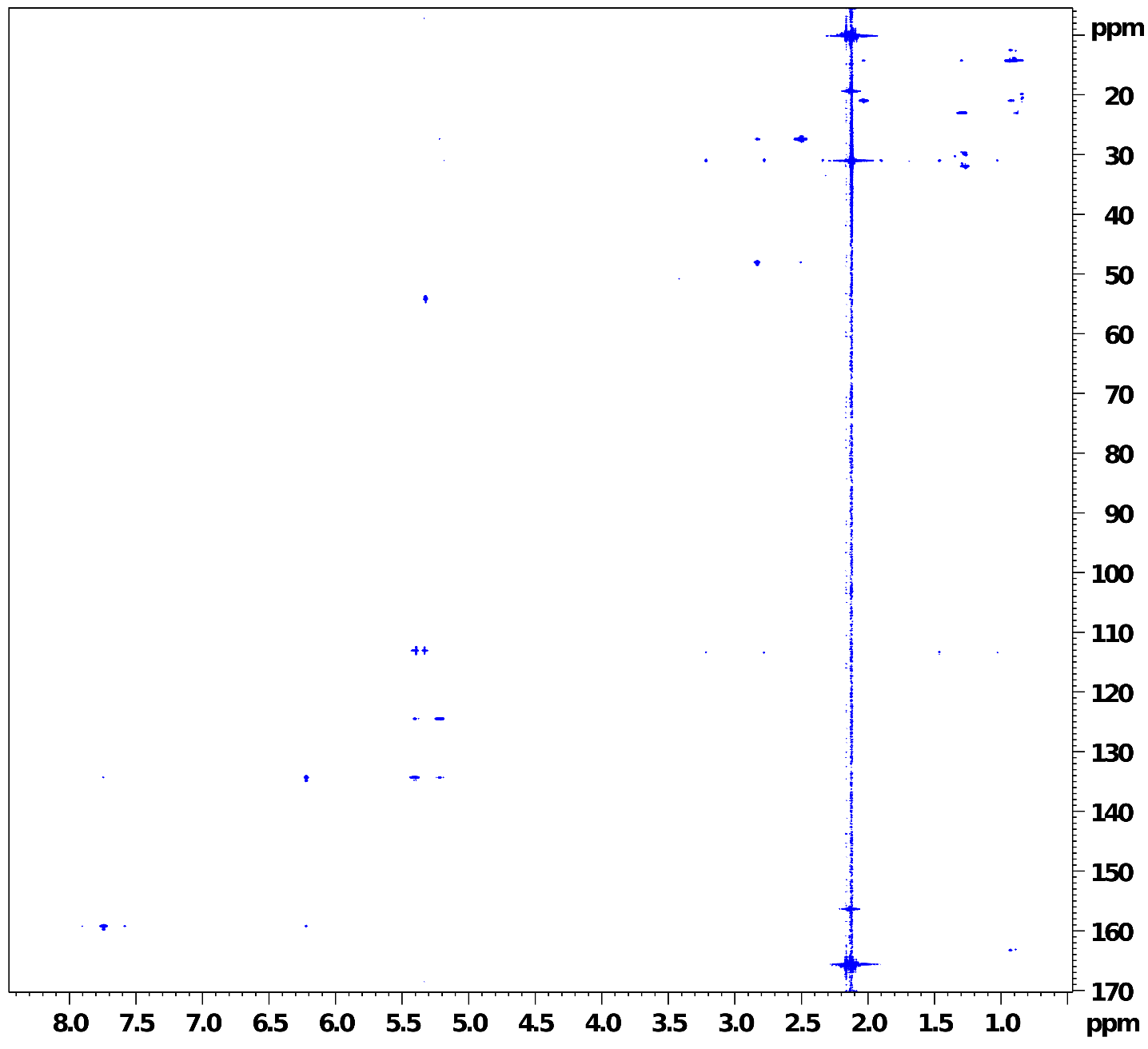


**Figure S10.** ^1^H,^13^C HSQC of compound **1**.


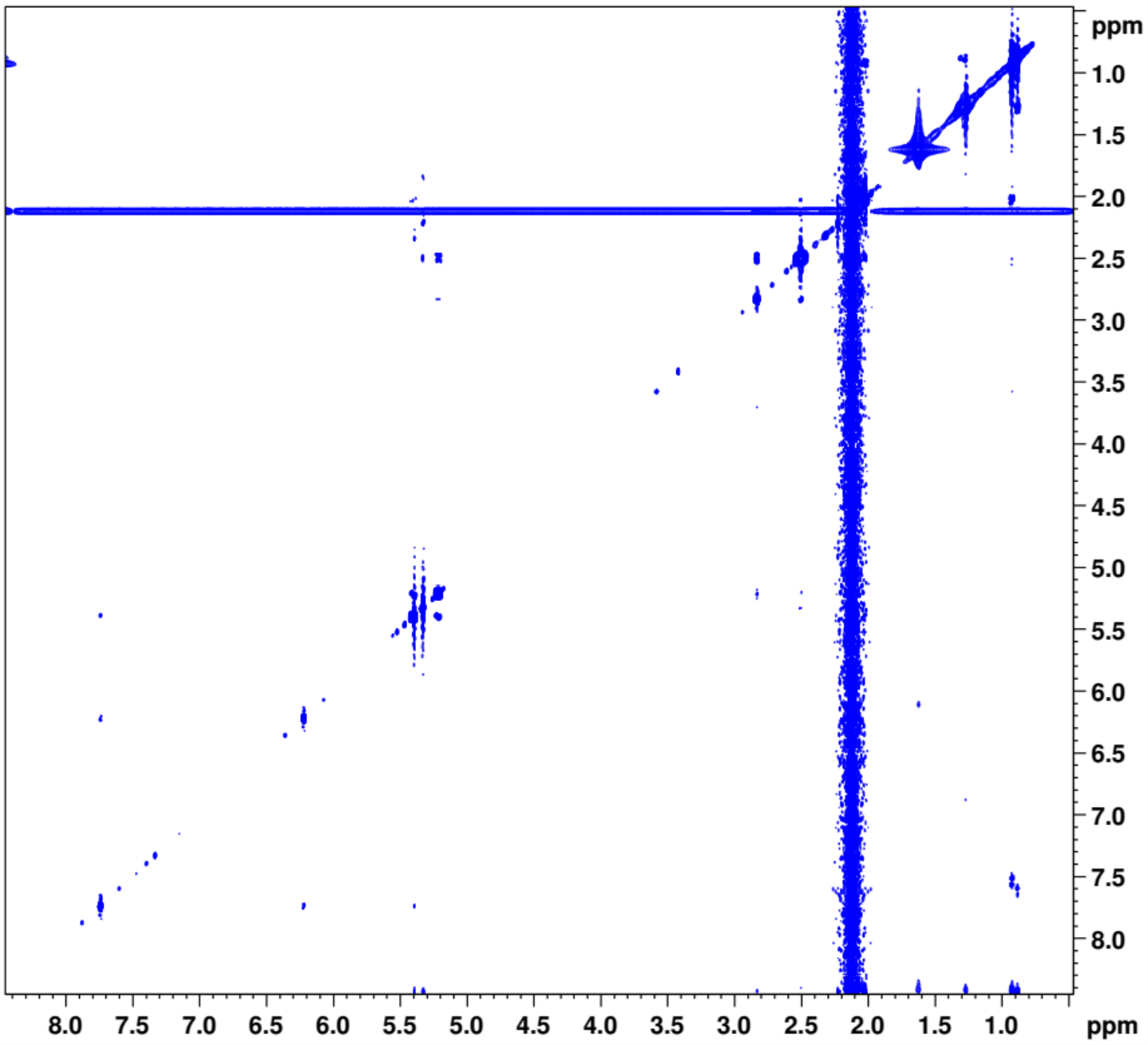


**Figure S11.** ^1^H,^1^H NOESY of compound **1**.


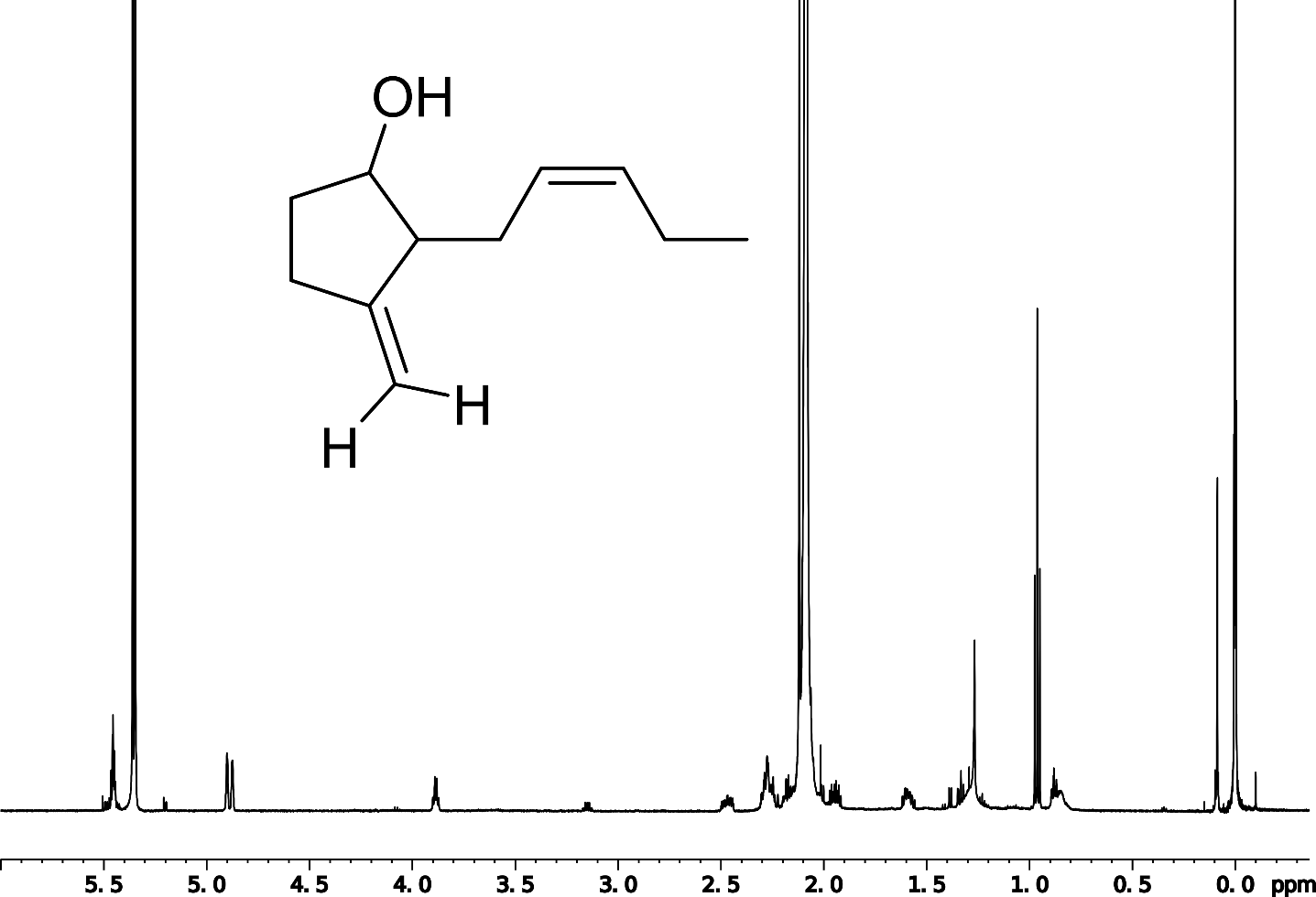


**Figure S12.** ^1^H NMR of compound **2**.


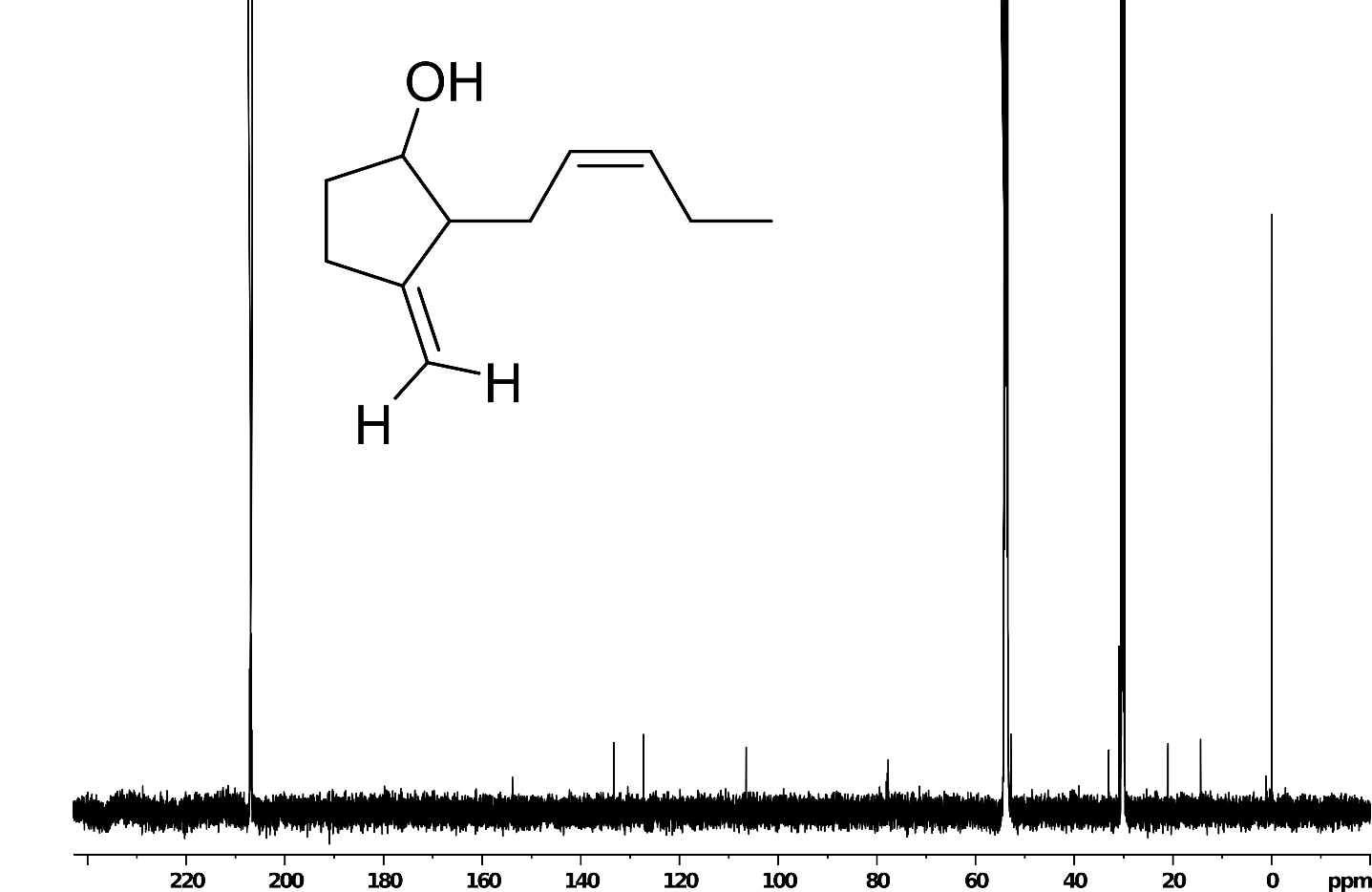


**Figure S13.** ^13^C NMR of compound **2**.


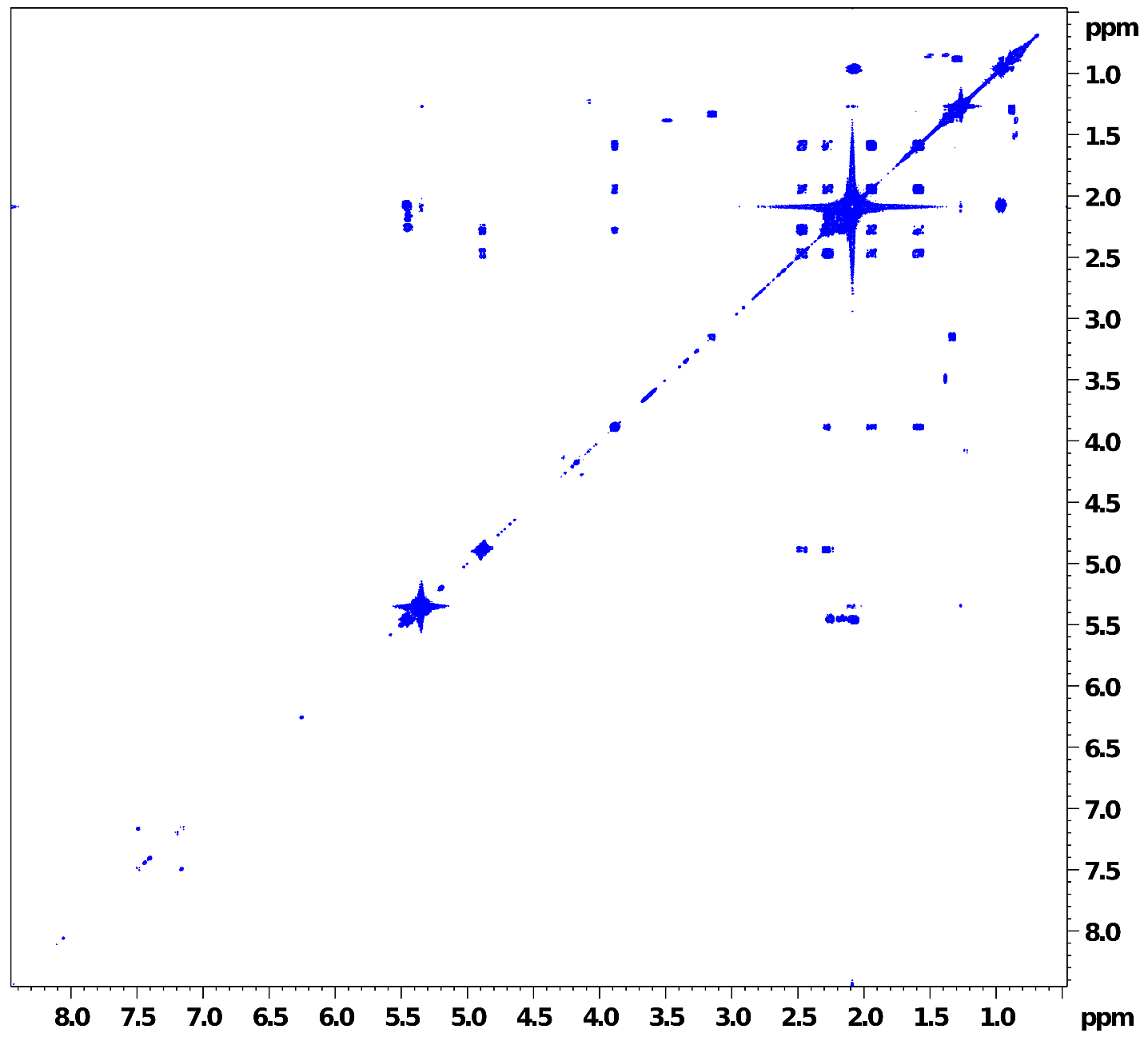


**Figure S14.** ^1^H,^1^H COSY of compound **2**.


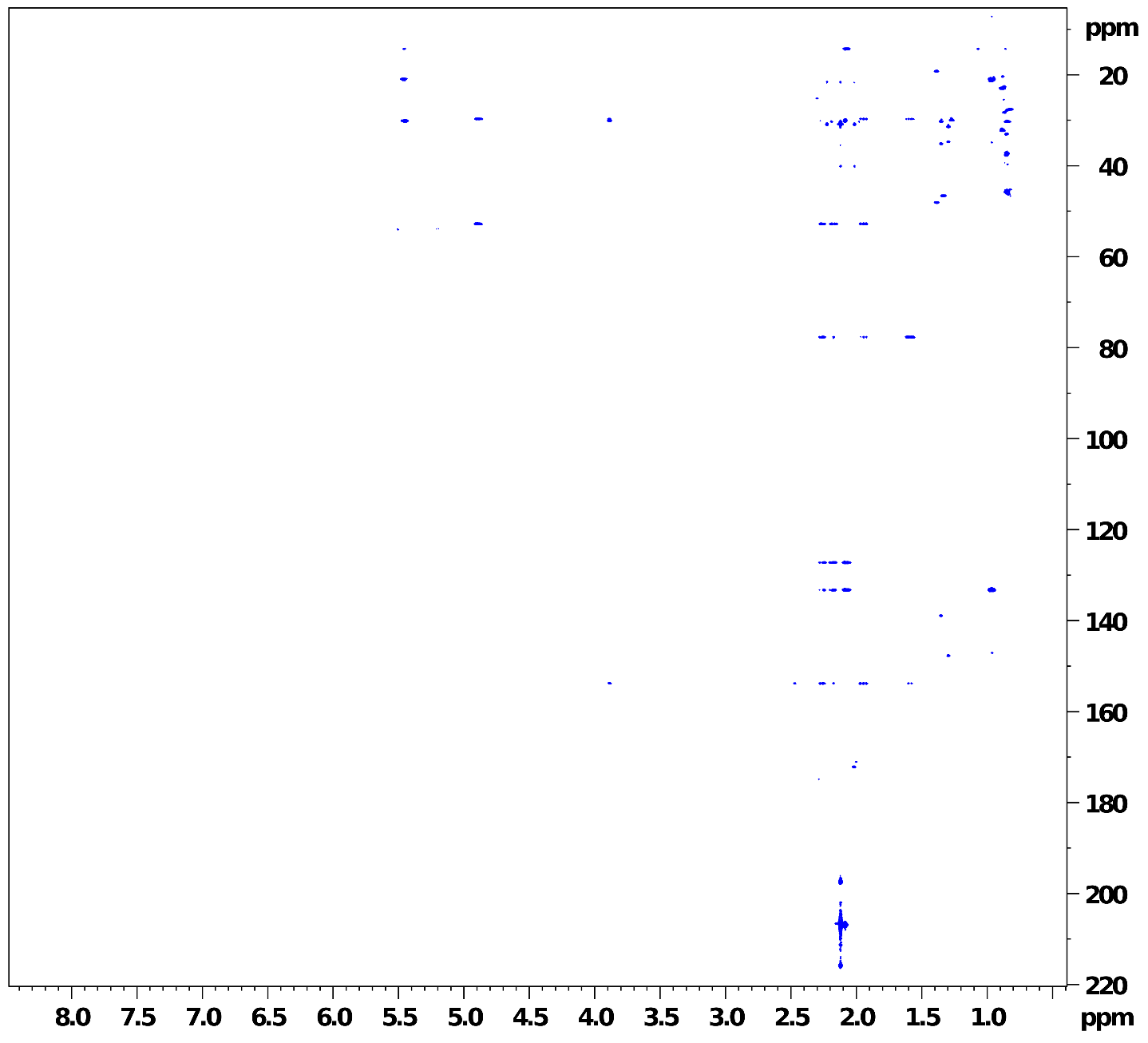


**Figure S15.** ^1^H,^13^C HMBC of compound **2**.


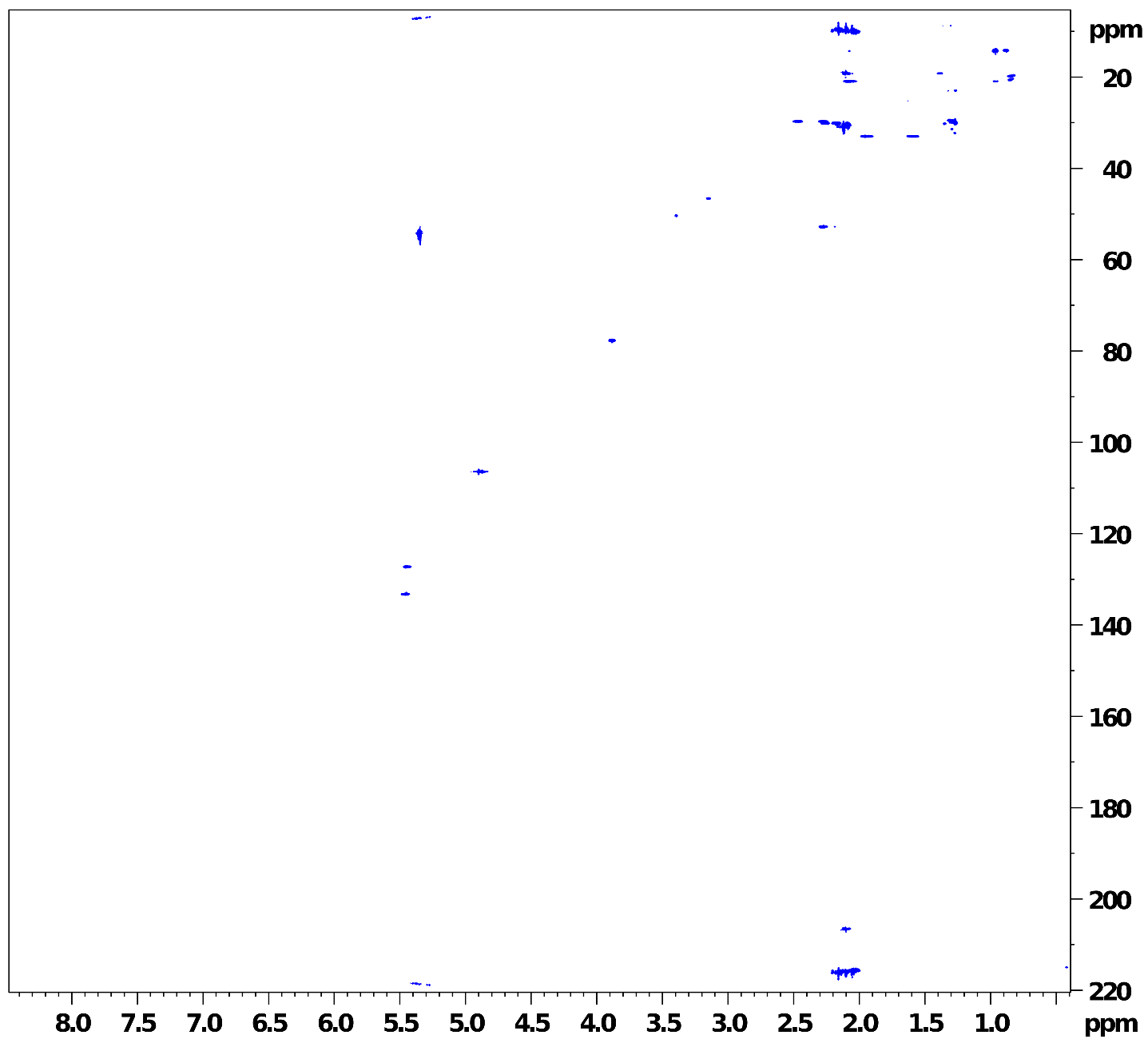


**Figure S16.** ^1^H,^13^C HSQC of compound **2**.


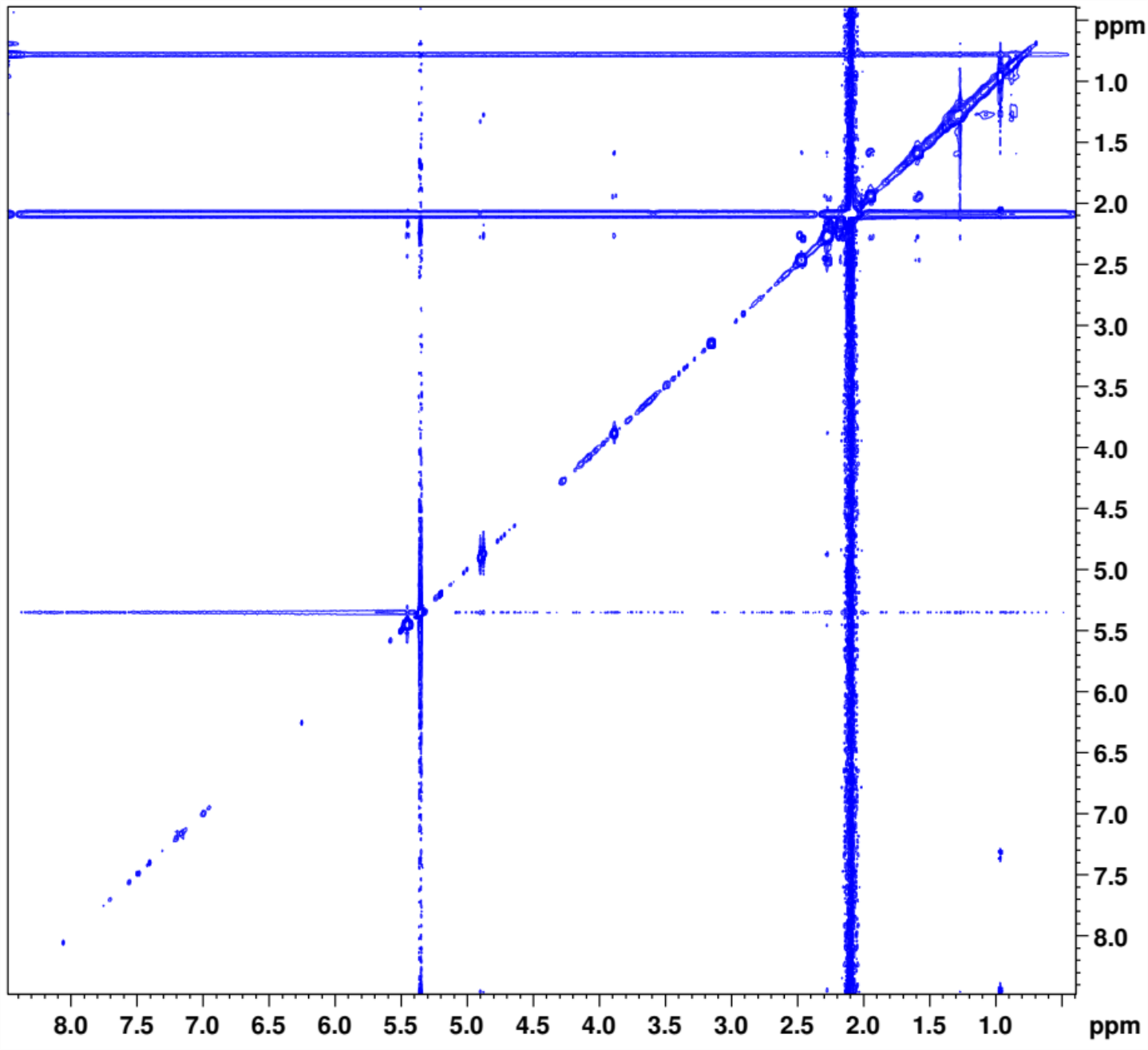


**Figure S17.** ^1^H,^1^H NOESY of compound **2**.


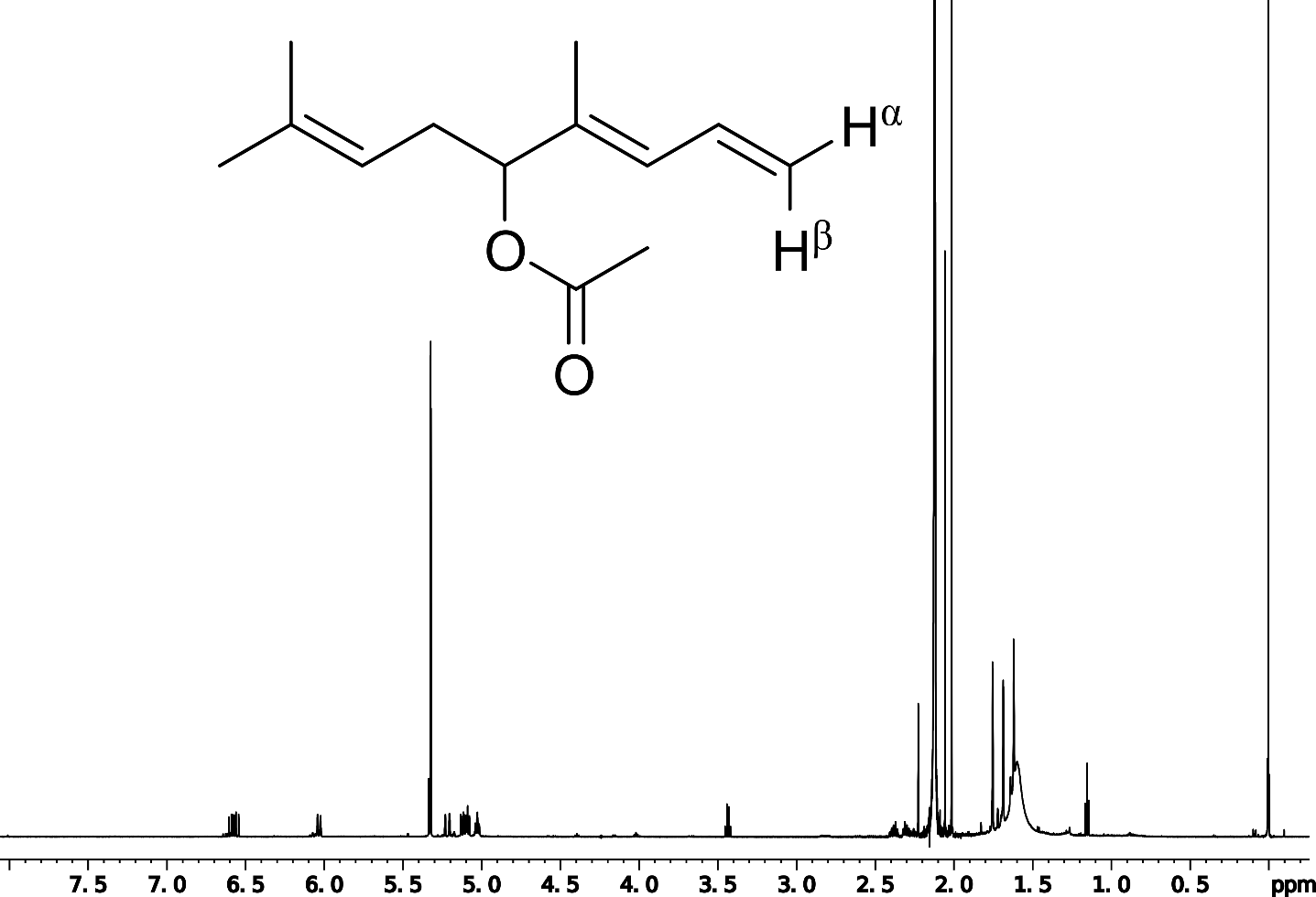


**Figure S18.** ^1^H NMR of compound **4**.


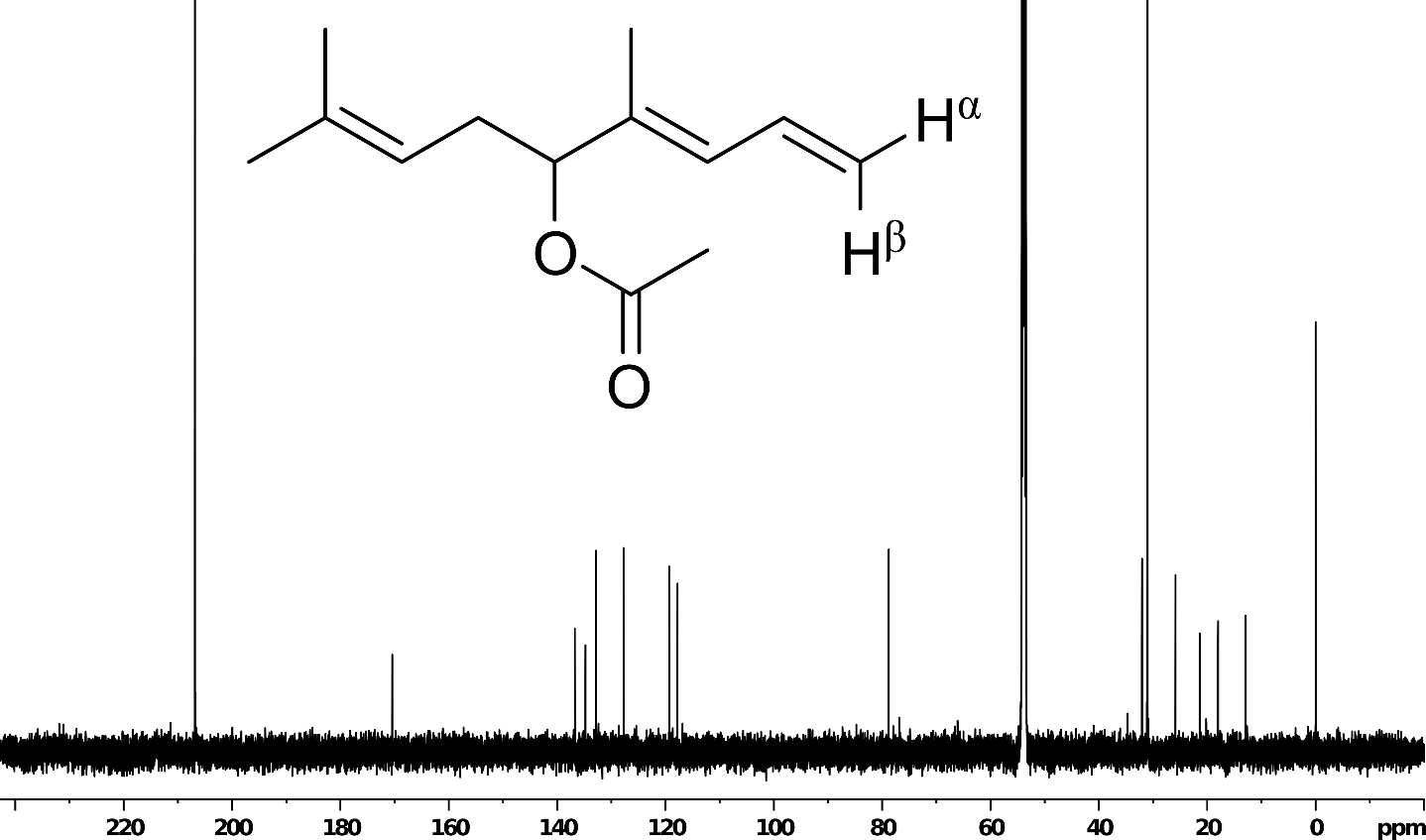


**Figure S19.** ^13^C NMR of compound **4**.


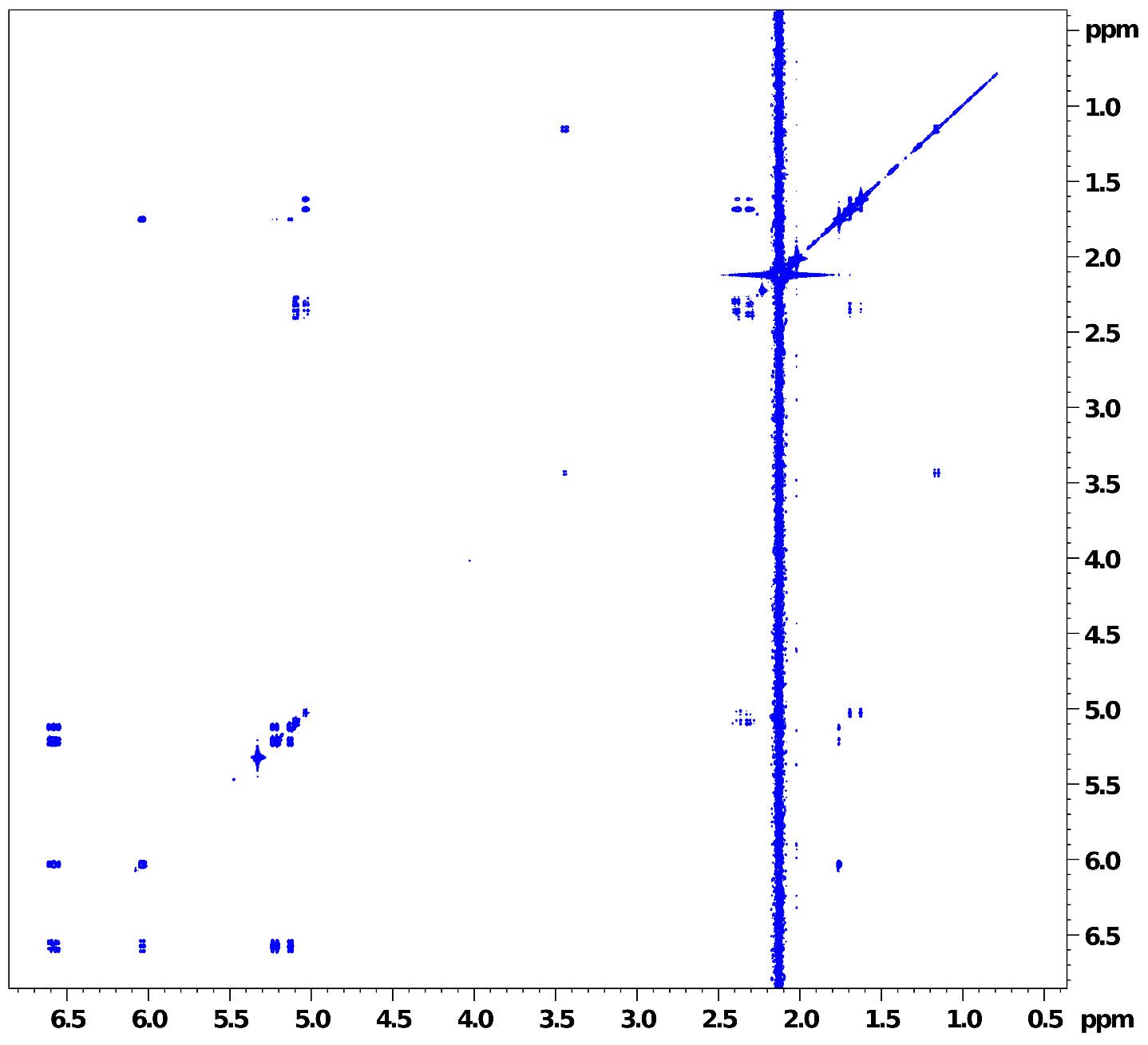


**Figure S20.** ^1^H,^1^H COSY of compound **4**.


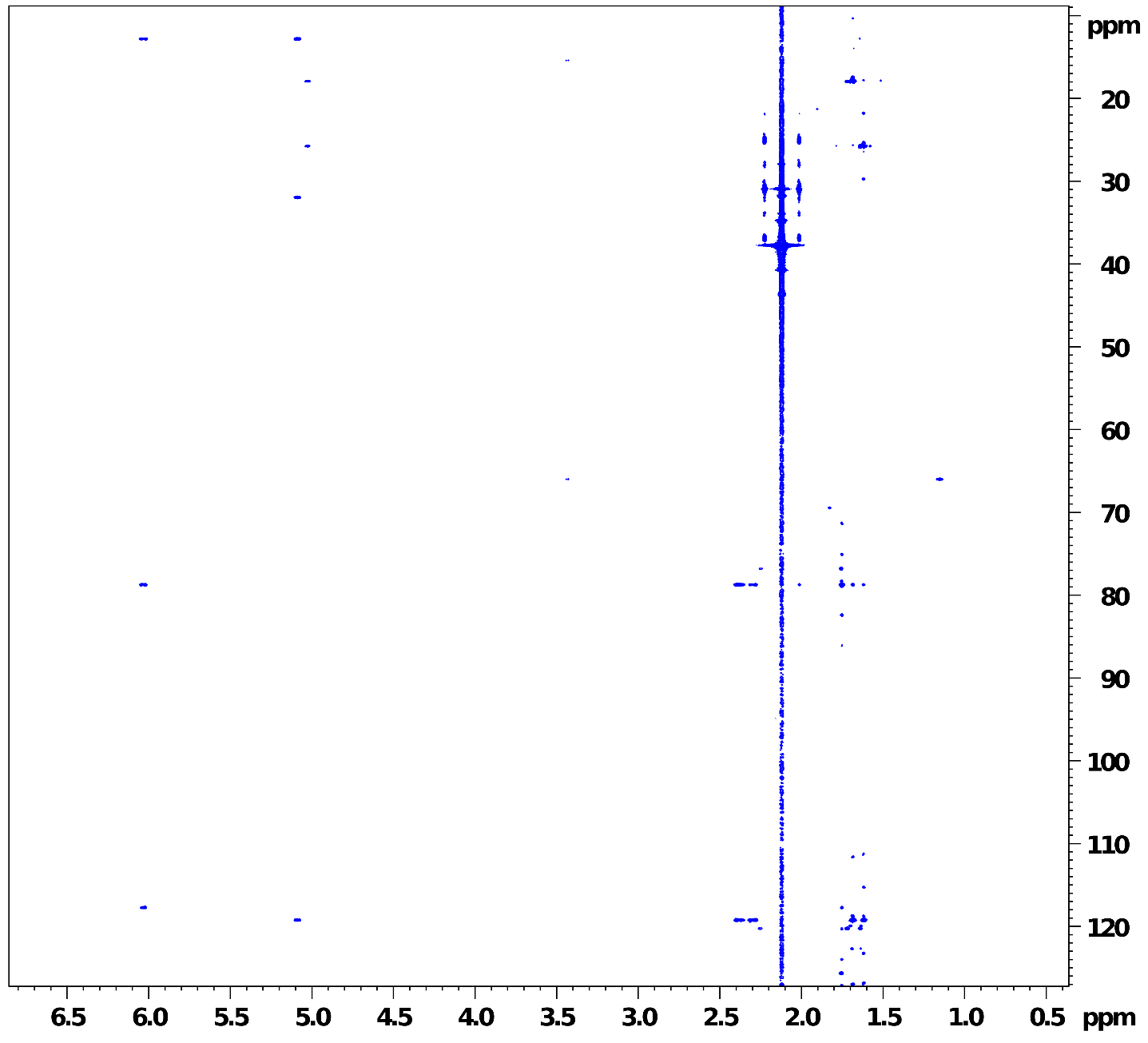


**Figure S21.** ^1^H,^13^C HMBC of compound **4**.


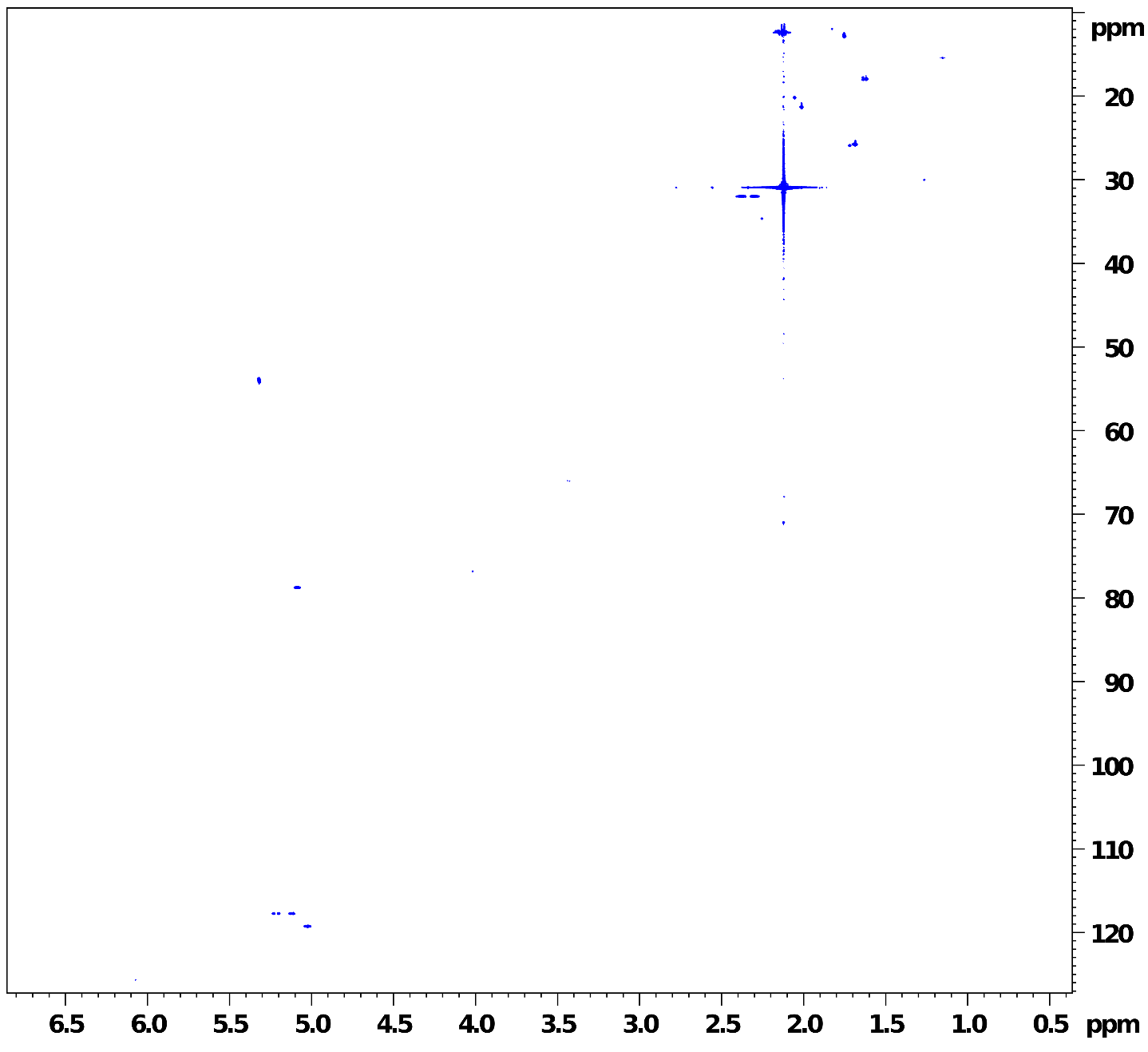


**Figure S22.** ^1^H,^13^C HSQC of compound **4**.
